# Supplementary figures and images for: SenSet defines cell-type specific senescence signatures in the aged human lung
Source: EMBO J. 2026 Apr 10;45(10):3589–638. doi: 10.1038/s44318-026-00762-8 (PMC13187336; doi:10.1038/s44318-026-00762-8)

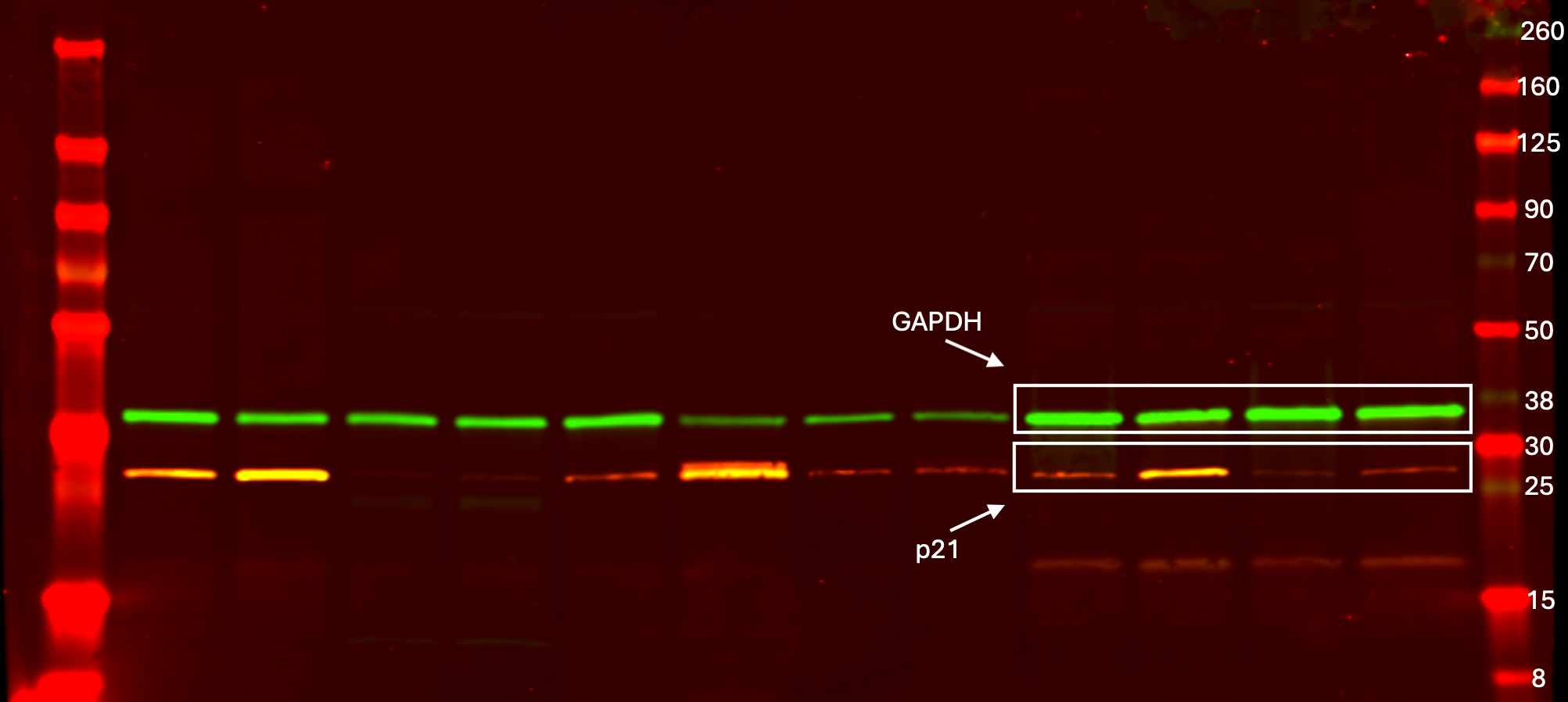

Supplement: Supplementary file 4 — Source data Fig. 5 [file 44318_2026_762_MOESM4_ESM.zip › Figure 5/5G/GAPDH and p21 fluo.tif]

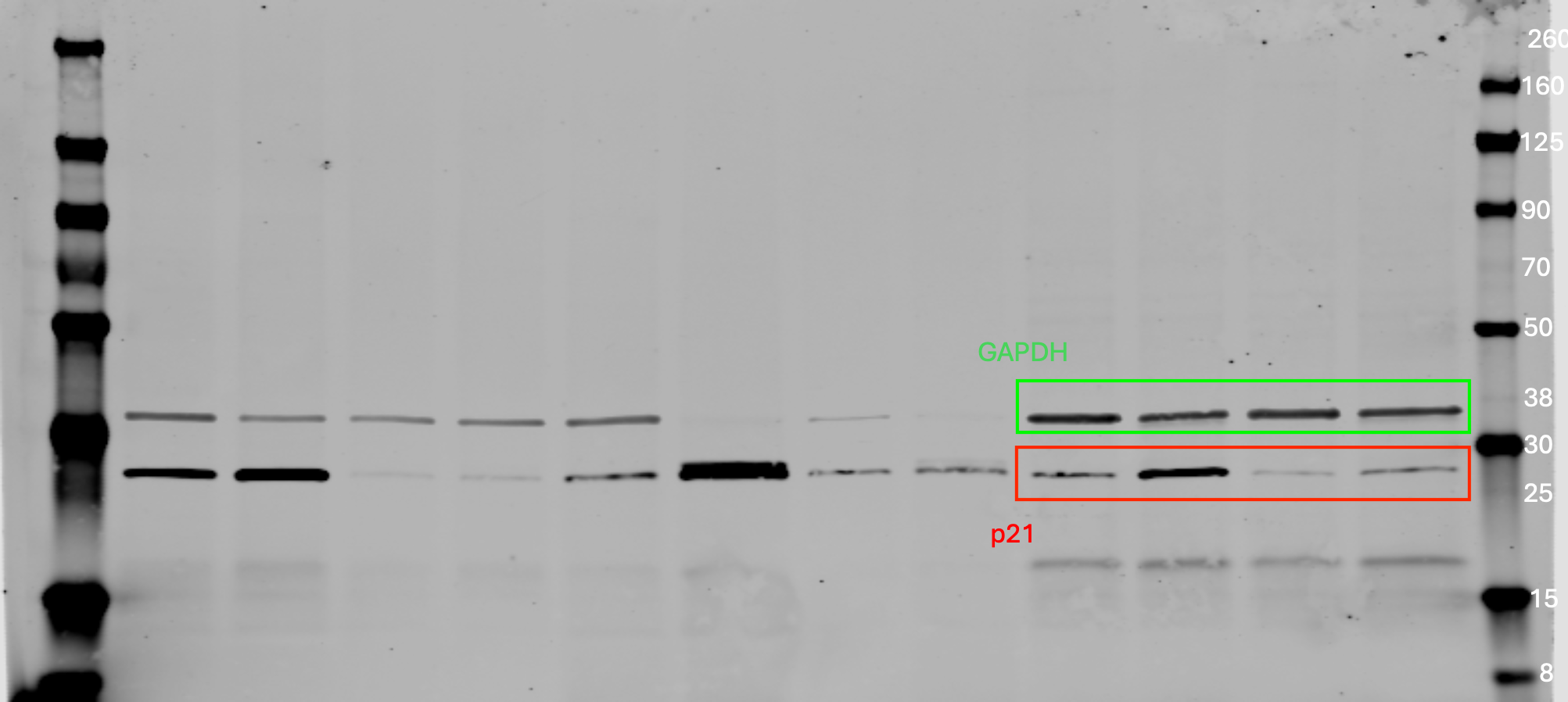

Supplement: Supplementary file 4 — Source data Fig. 5 [file 44318_2026_762_MOESM4_ESM.zip › Figure 5/5G/GAPDH and p21 grey.tif]

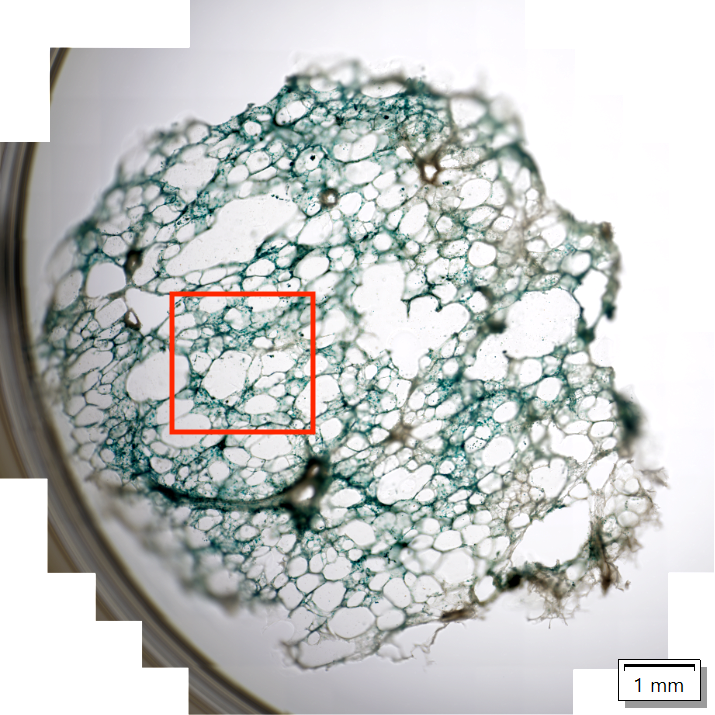

Supplement: Supplementary file 4 — Source data Fig. 5 [file 44318_2026_762_MOESM4_ESM.zip › Figure 5/5C/beta galactosidase BLEO.tif]

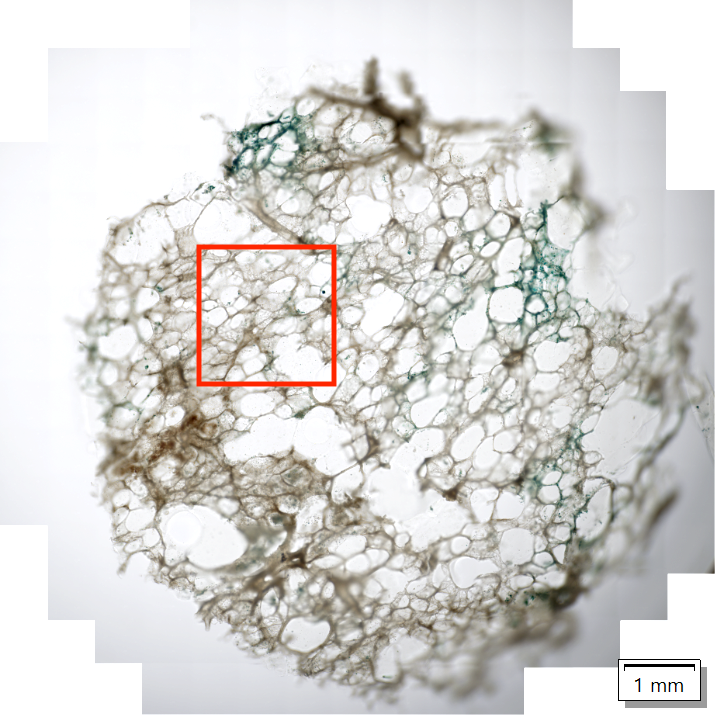

Supplement: Supplementary file 4 — Source data Fig. 5 [file 44318_2026_762_MOESM4_ESM.zip › Figure 5/5C/beta galactosidase CTL.tif]

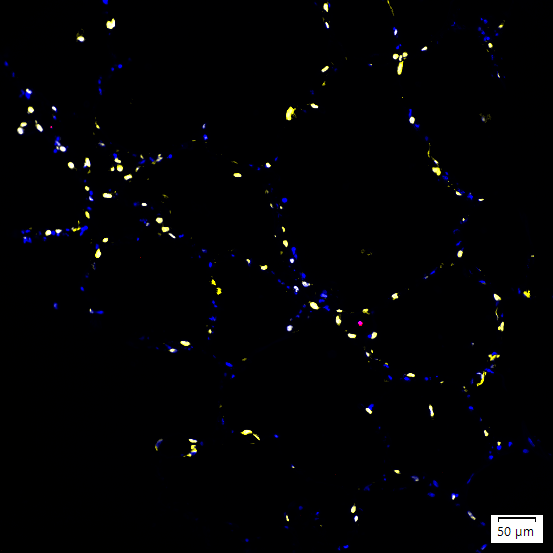

Supplement: Supplementary file 4 — Source data Fig. 5 [file 44318_2026_762_MOESM4_ESM.zip › Figure 5/5D/Bleo_merge.tif]

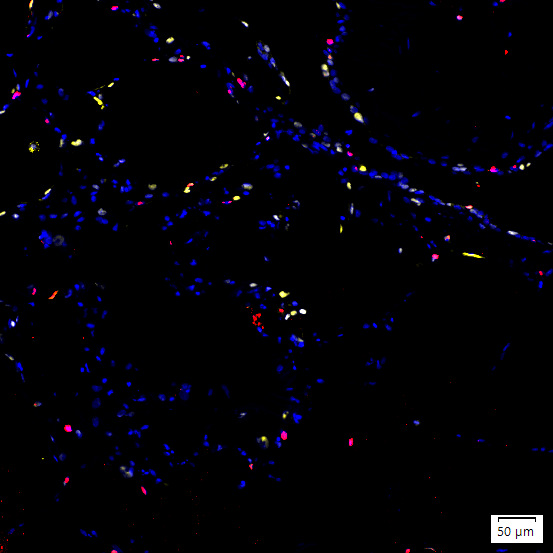

Supplement: Supplementary file 4 — Source data Fig. 5 [file 44318_2026_762_MOESM4_ESM.zip › Figure 5/5D/Bleo control_merge.tif]

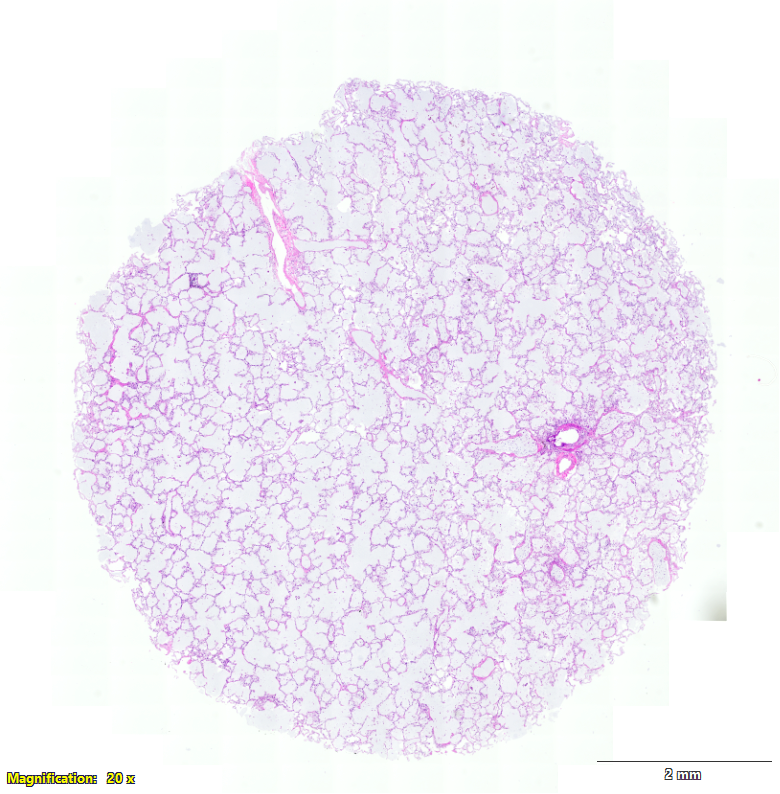

Supplement: Supplementary file 4 — Source data Fig. 5 [file 44318_2026_762_MOESM4_ESM.zip › Figure 5/5B/Bleo_H&N.tif]

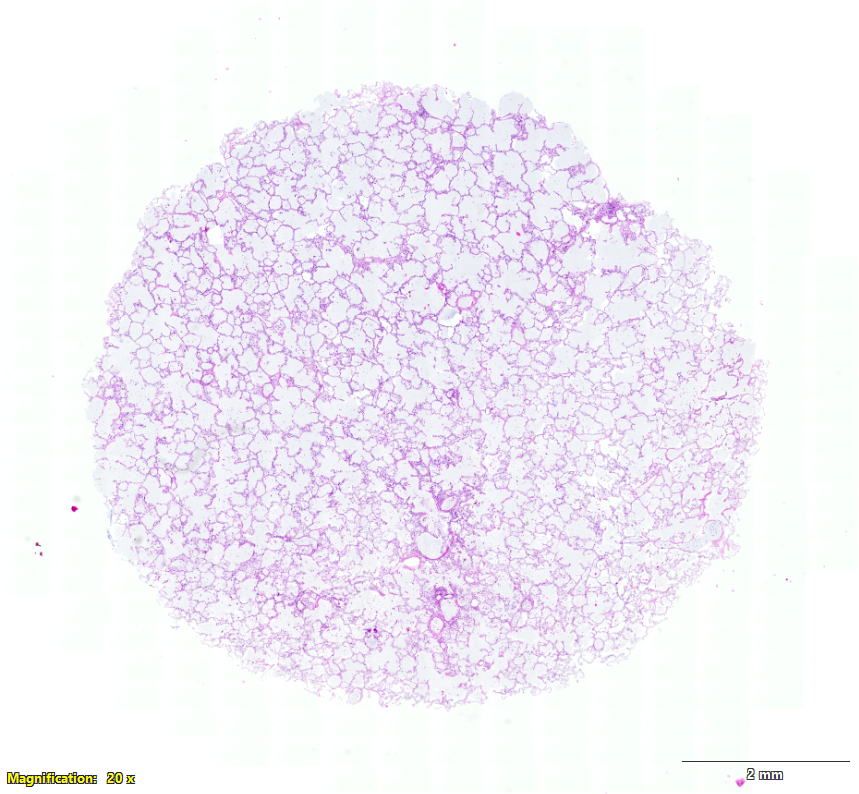

Supplement: Supplementary file 4 — Source data Fig. 5 [file 44318_2026_762_MOESM4_ESM.zip › Figure 5/5B/Bleo control_H&N.tif]

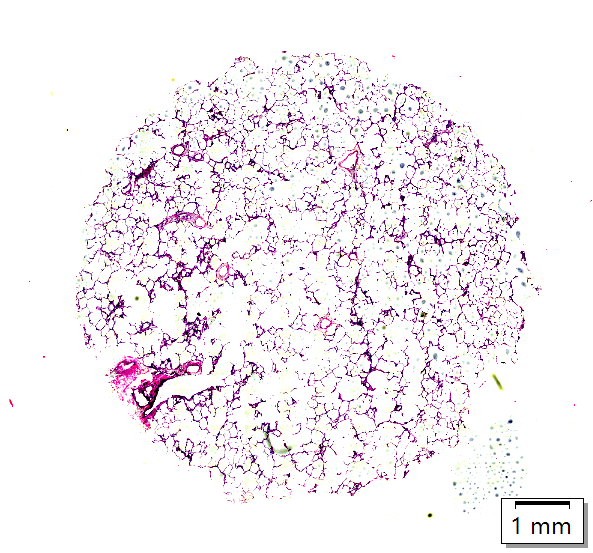

Supplement: Supplementary file 11 — Figure EV8 Source Data [file 44318_2026_762_MOESM11_ESM.zip › Figure EV9/EV9A H&E/Bleo.tif]

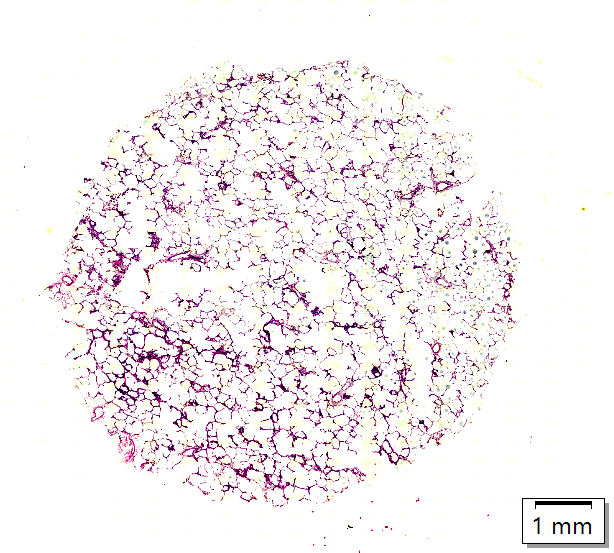

Supplement: Supplementary file 11 — Figure EV8 Source Data [file 44318_2026_762_MOESM11_ESM.zip › Figure EV9/EV9A H&E/Bleo control.tif]

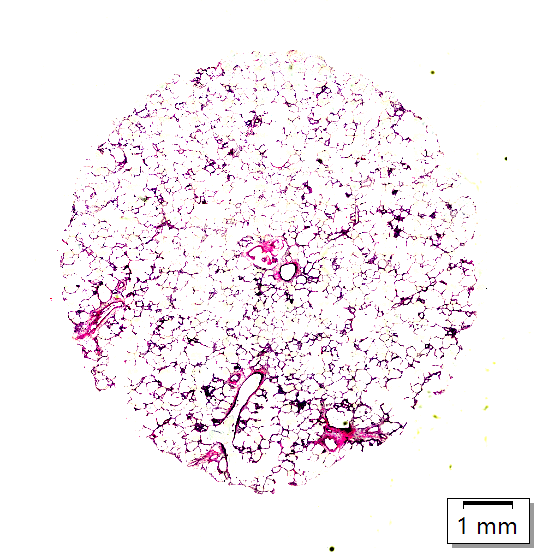

Supplement: Supplementary file 11 — Figure EV8 Source Data [file 44318_2026_762_MOESM11_ESM.zip › Figure EV9/EV9A H&E/Doxo control.tif]

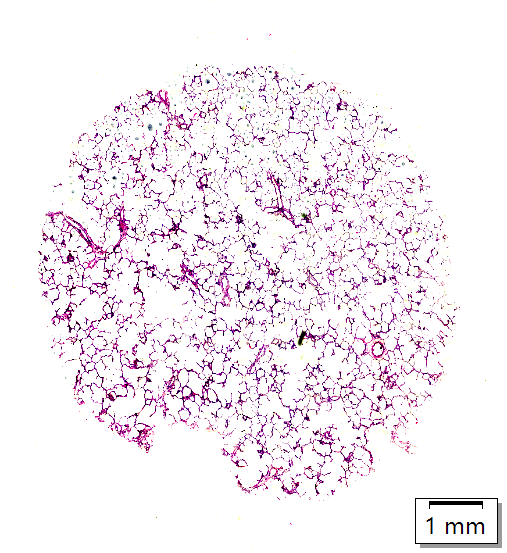

Supplement: Supplementary file 11 — Figure EV8 Source Data [file 44318_2026_762_MOESM11_ESM.zip › Figure EV9/EV9A H&E/Doxo.tif]

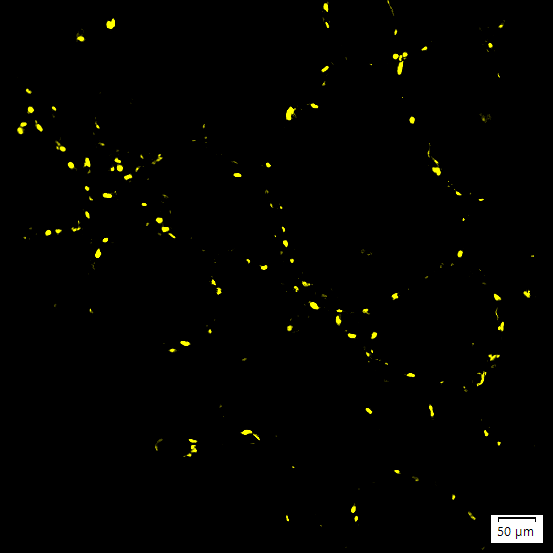

Supplement: Supplementary file 11 — Figure EV8 Source Data [file 44318_2026_762_MOESM11_ESM.zip › Figure EV9/EV9B fluo images/Bleo_p21.tif]

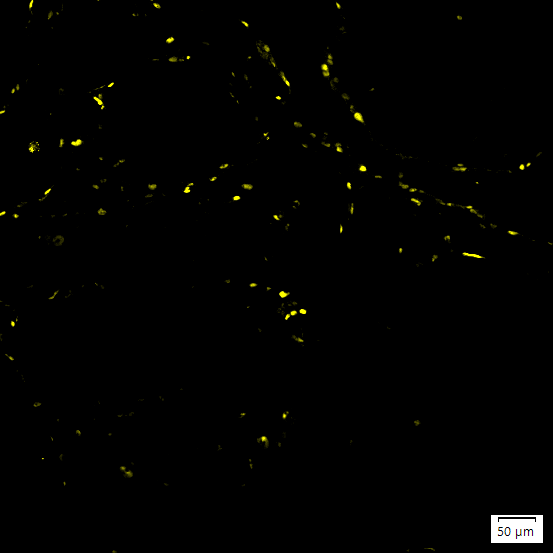

Supplement: Supplementary file 11 — Figure EV8 Source Data [file 44318_2026_762_MOESM11_ESM.zip › Figure EV9/EV9B fluo images/Bleo control_p21.tif]

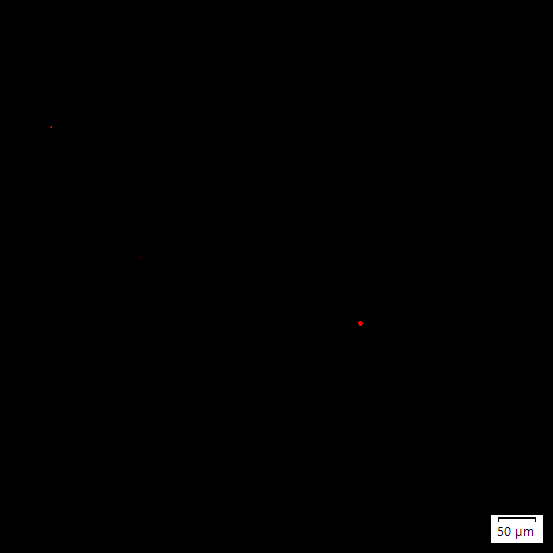

Supplement: Supplementary file 11 — Figure EV8 Source Data [file 44318_2026_762_MOESM11_ESM.zip › Figure EV9/EV9B fluo images/Bleo_ki67.tif]

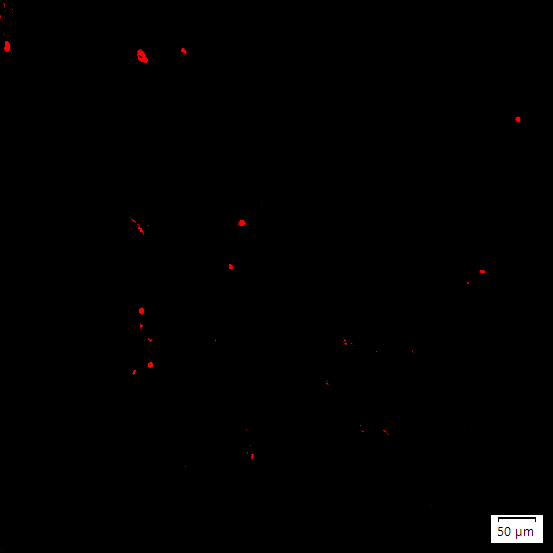

Supplement: Supplementary file 11 — Figure EV8 Source Data [file 44318_2026_762_MOESM11_ESM.zip › Figure EV9/EV9B fluo images/Doxo control_ki67.tif]

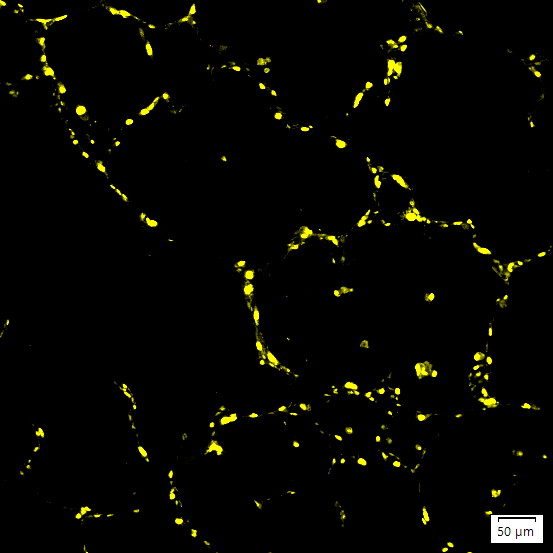

Supplement: Supplementary file 11 — Figure EV8 Source Data [file 44318_2026_762_MOESM11_ESM.zip › Figure EV9/EV9B fluo images/Doxo_p21.tif]

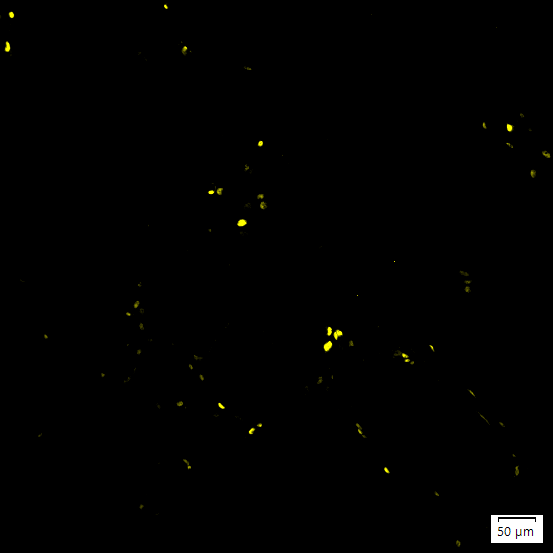

Supplement: Supplementary file 11 — Figure EV8 Source Data [file 44318_2026_762_MOESM11_ESM.zip › Figure EV9/EV9B fluo images/Doxo control_p21.tif]

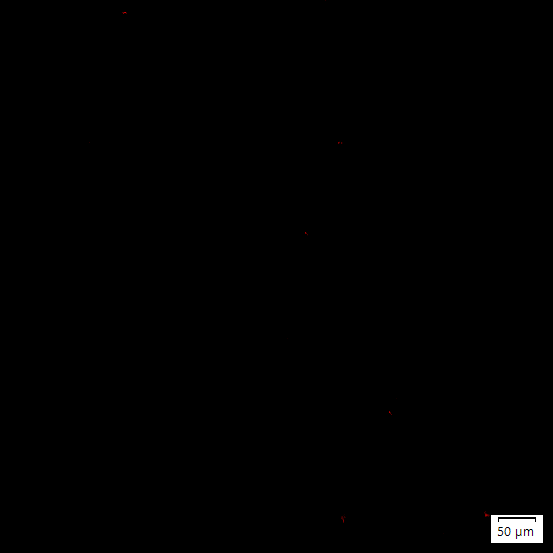

Supplement: Supplementary file 11 — Figure EV8 Source Data [file 44318_2026_762_MOESM11_ESM.zip › Figure EV9/EV9B fluo images/Doxo_ki67.tif]

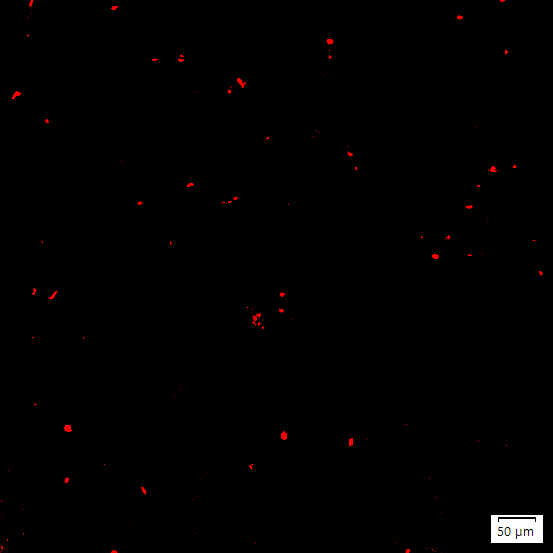

Supplement: Supplementary file 11 — Figure EV8 Source Data [file 44318_2026_762_MOESM11_ESM.zip › Figure EV9/EV9B fluo images/Bleo control_ki67.tif]

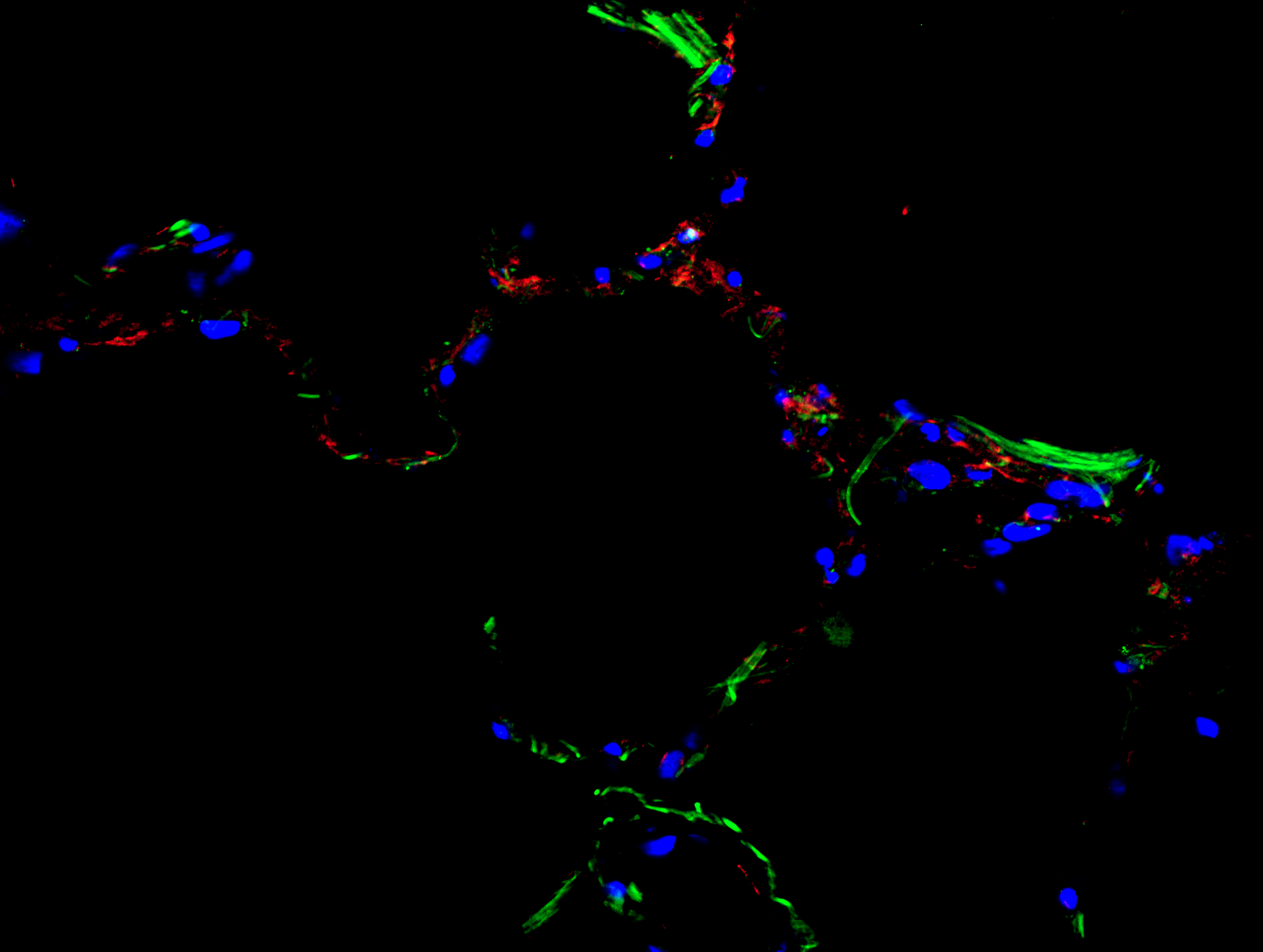

Supplement: Supplementary file 12 — Figure EV9 Source Data [file 44318_2026_762_MOESM12_ESM.zip › Figure EV10/EV10F/IF_hPCLS_Control_aSMA_Fibronectin_DAPI/hPCLS_Control_aSMA_Fibronectin_DAPI_Merge.tif]

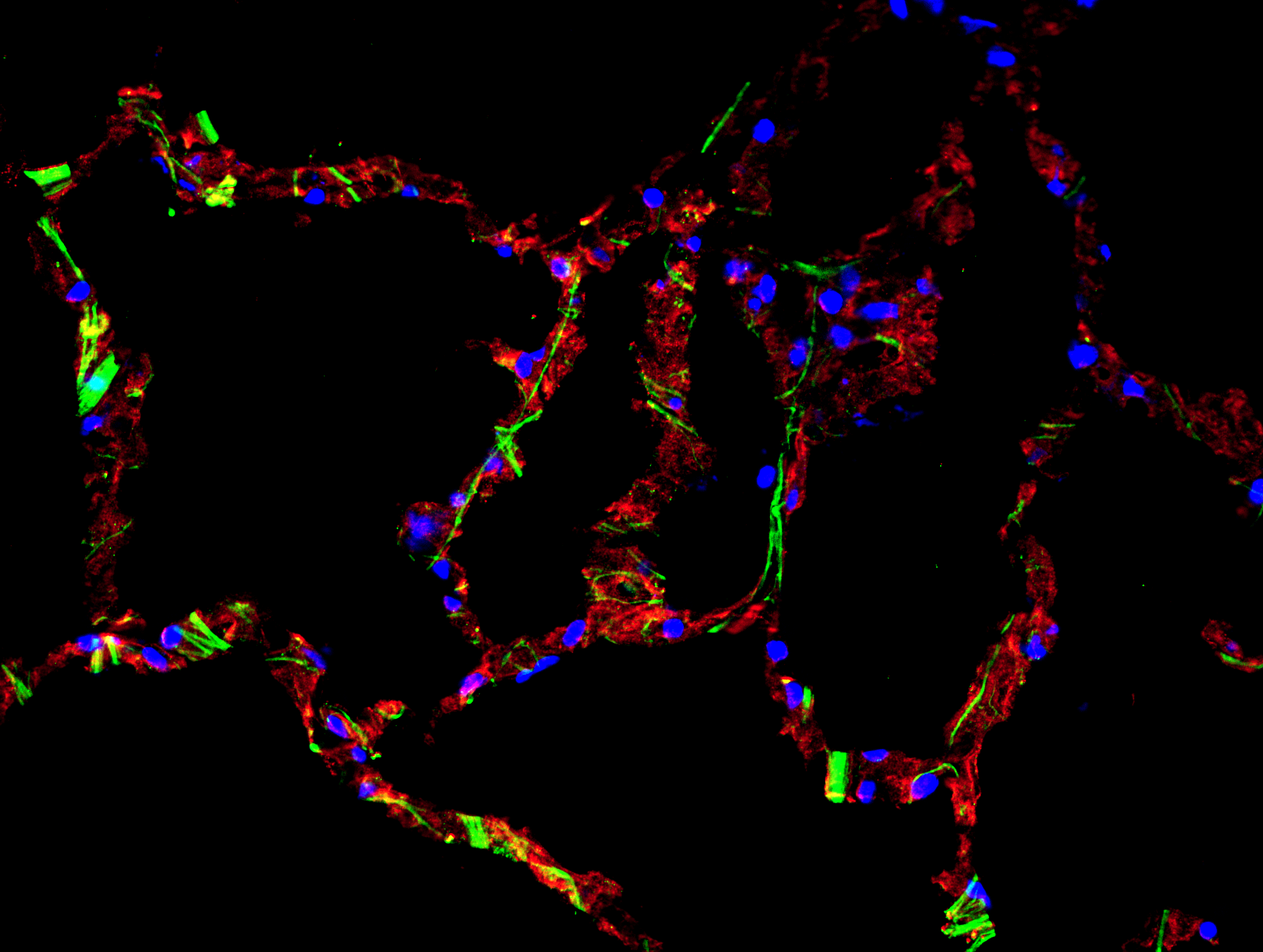

Supplement: Supplementary file 12 — Figure EV9 Source Data [file 44318_2026_762_MOESM12_ESM.zip › Figure EV10/EV10F/IF_hPCLS_Bleo_aSMA_Fibronectin_DAPI/hPCLS_Bleo_aSMA_Fibronectin_DAPI_Merge.tif]

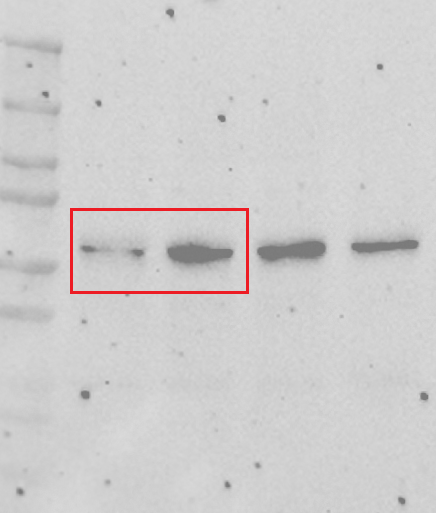

Supplement: Supplementary file 12 — Figure EV9 Source Data [file 44318_2026_762_MOESM12_ESM.zip › Figure EV10/EV10G/hPCLS_aSMA_Ladder.png]

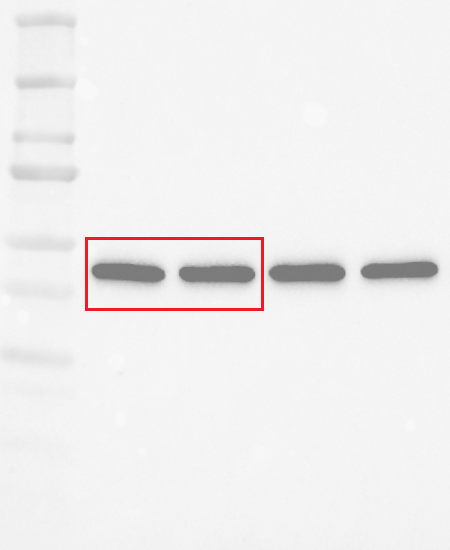

Supplement: Supplementary file 12 — Figure EV9 Source Data [file 44318_2026_762_MOESM12_ESM.zip › Figure EV10/EV10G/hPCLS_B-actin_Ladder.png]

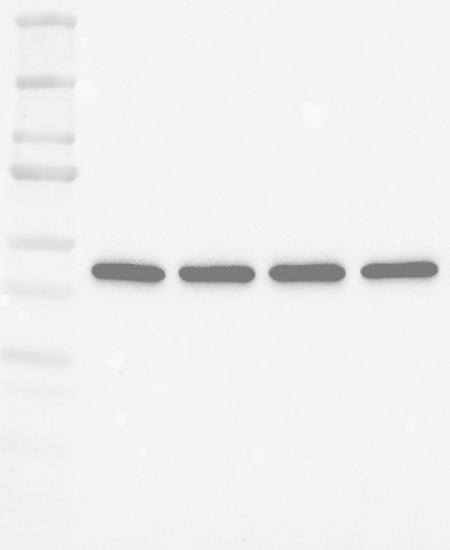

Supplement: Supplementary file 12 — Figure EV9 Source Data [file 44318_2026_762_MOESM12_ESM.zip › Figure EV10/EV10G/hPCLS_B-actin_Ladder.tif]

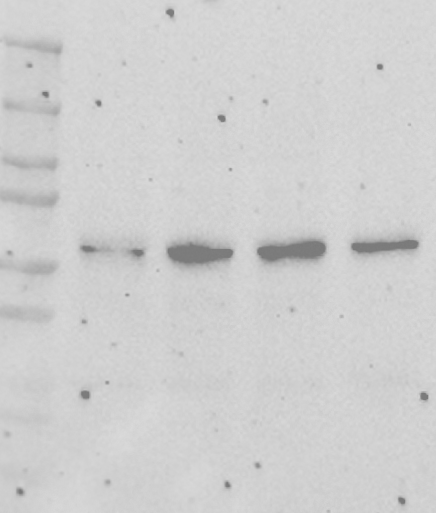

Supplement: Supplementary file 12 — Figure EV9 Source Data [file 44318_2026_762_MOESM12_ESM.zip › Figure EV10/EV10G/hPCLS_aSMA_Ladder.tif]

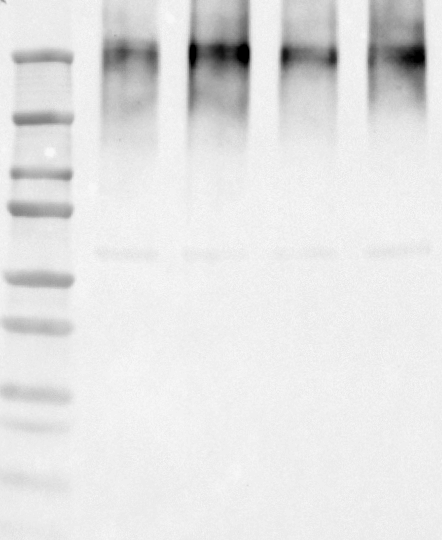

Supplement: Supplementary file 12 — Figure EV9 Source Data [file 44318_2026_762_MOESM12_ESM.zip › Figure EV10/EV10G/hPCLS_Fibronectin_Ladder.tif]

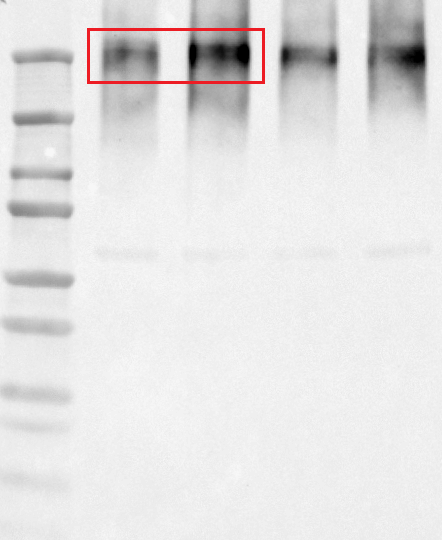

Supplement: Supplementary file 12 — Figure EV9 Source Data [file 44318_2026_762_MOESM12_ESM.zip › Figure EV10/EV10G/hPCLS_Fibronectin_Ladder.png]

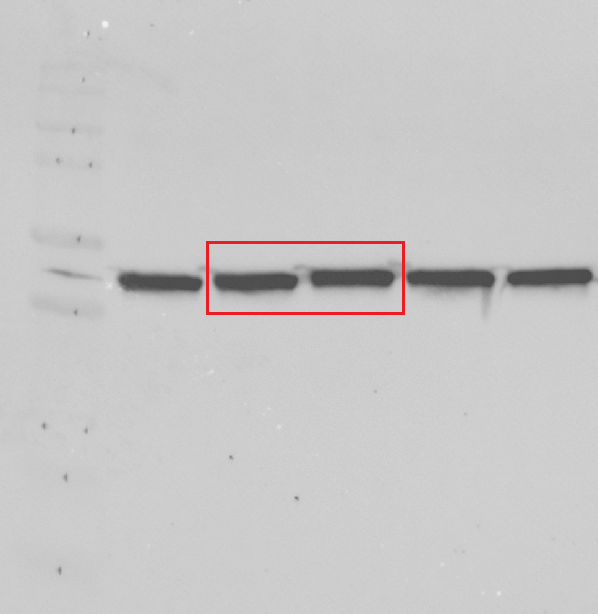

Supplement: Supplementary file 12 — Figure EV9 Source Data [file 44318_2026_762_MOESM12_ESM.zip › Figure EV10/EV10B/hPCLS_bactin_Ladder.png]

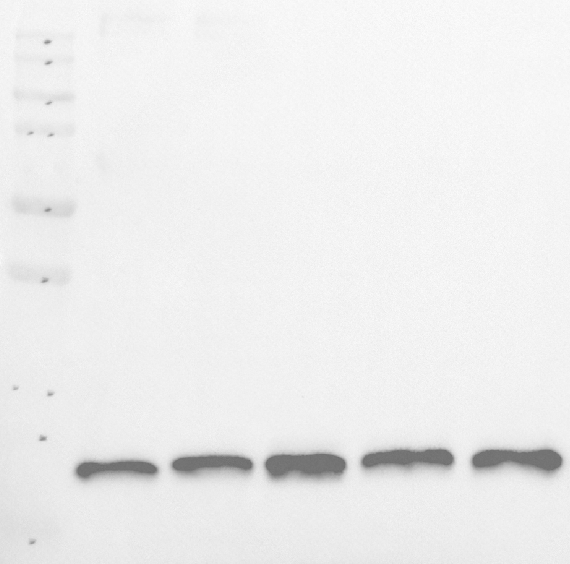

Supplement: Supplementary file 12 — Figure EV9 Source Data [file 44318_2026_762_MOESM12_ESM.zip › Figure EV10/EV10B/hPCLS_p16_Ladder.tif]

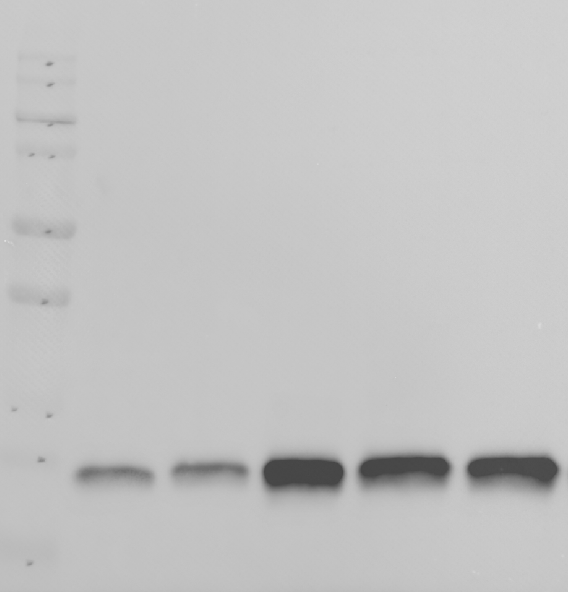

Supplement: Supplementary file 12 — Figure EV9 Source Data [file 44318_2026_762_MOESM12_ESM.zip › Figure EV10/EV10B/hPCLS_p21_Ladder.tif]

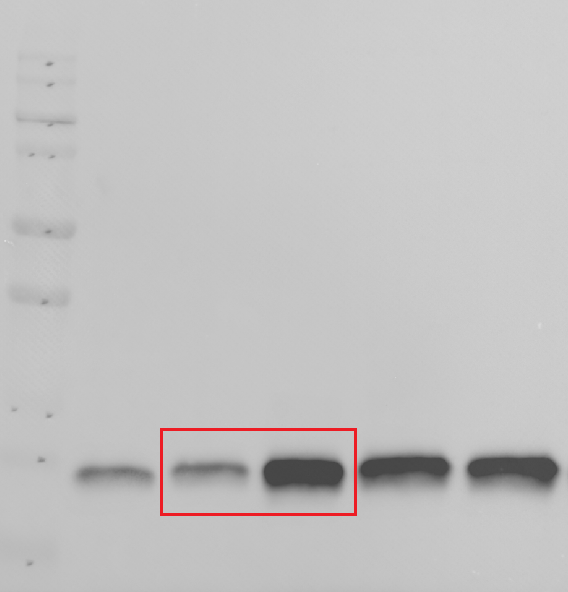

Supplement: Supplementary file 12 — Figure EV9 Source Data [file 44318_2026_762_MOESM12_ESM.zip › Figure EV10/EV10B/hPCLS_p21_Ladder.png]

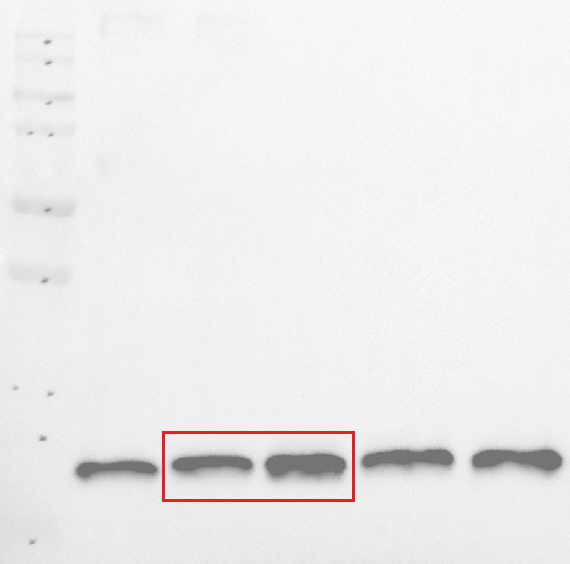

Supplement: Supplementary file 12 — Figure EV9 Source Data [file 44318_2026_762_MOESM12_ESM.zip › Figure EV10/EV10B/hPCLS_p16_Ladder.png]

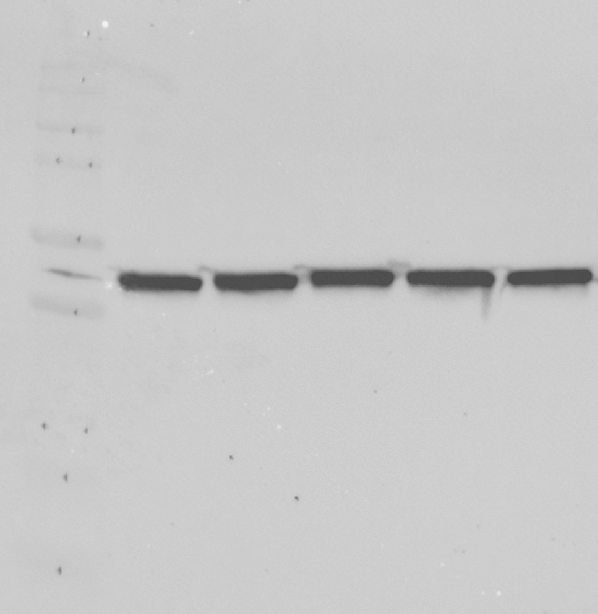

Supplement: Supplementary file 12 — Figure EV9 Source Data [file 44318_2026_762_MOESM12_ESM.zip › Figure EV10/EV10B/hPCLS_bactin_Ladder.tif]
