# Supplementary material for: SenSet defines cell-type specific senescence signatures in the aged human lung
Source: EMBO J. 2026 Apr 10;45(10):3589–638. doi: 10.1038/s44318-026-00762-8 (PMC13187336; doi:10.1038/s44318-026-00762-8)
Supplement: Supplementary file 21 — Expanded View Figures [file 44318_2026_762_MOESM21_ESM.pdf]

Expanded View Figures

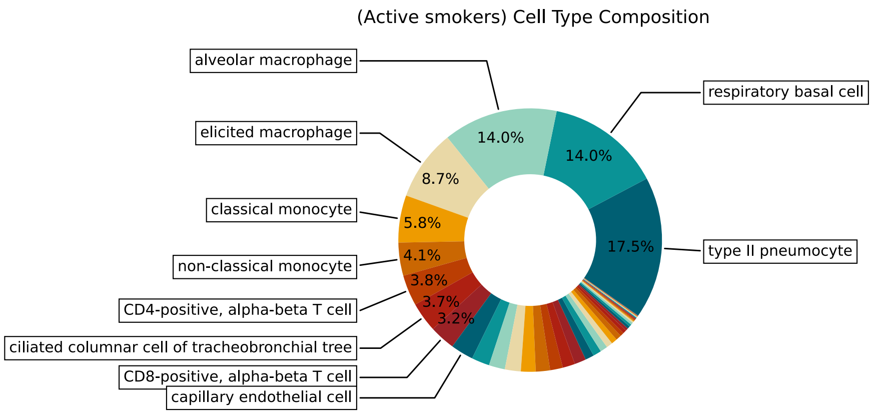

Figure EV1. Cell-type composition in active smokers.

Pie chart showing the relative contribution of each lung cell type in active smokers. Labels indicate the exact percentage contribution of each cell type.

© The Author(s)

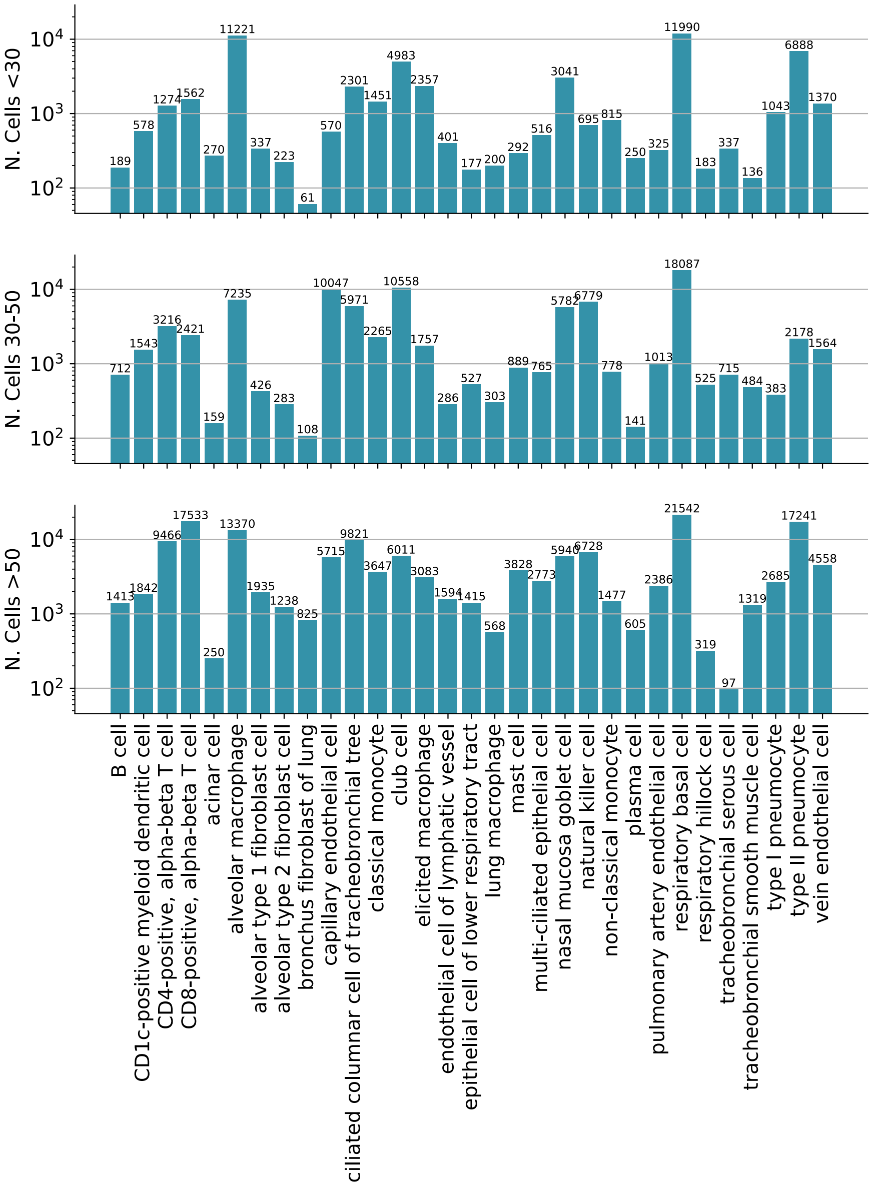

Figure EV2. Cell counts per cell type and age group in the HLCA (non-smokers only).

Bar charts showing the number of cells per cell type stratified by donor age group. Separate panels correspond to young (top), middle-aged (middle), and older (bottom) donors.

 © The Author(s)

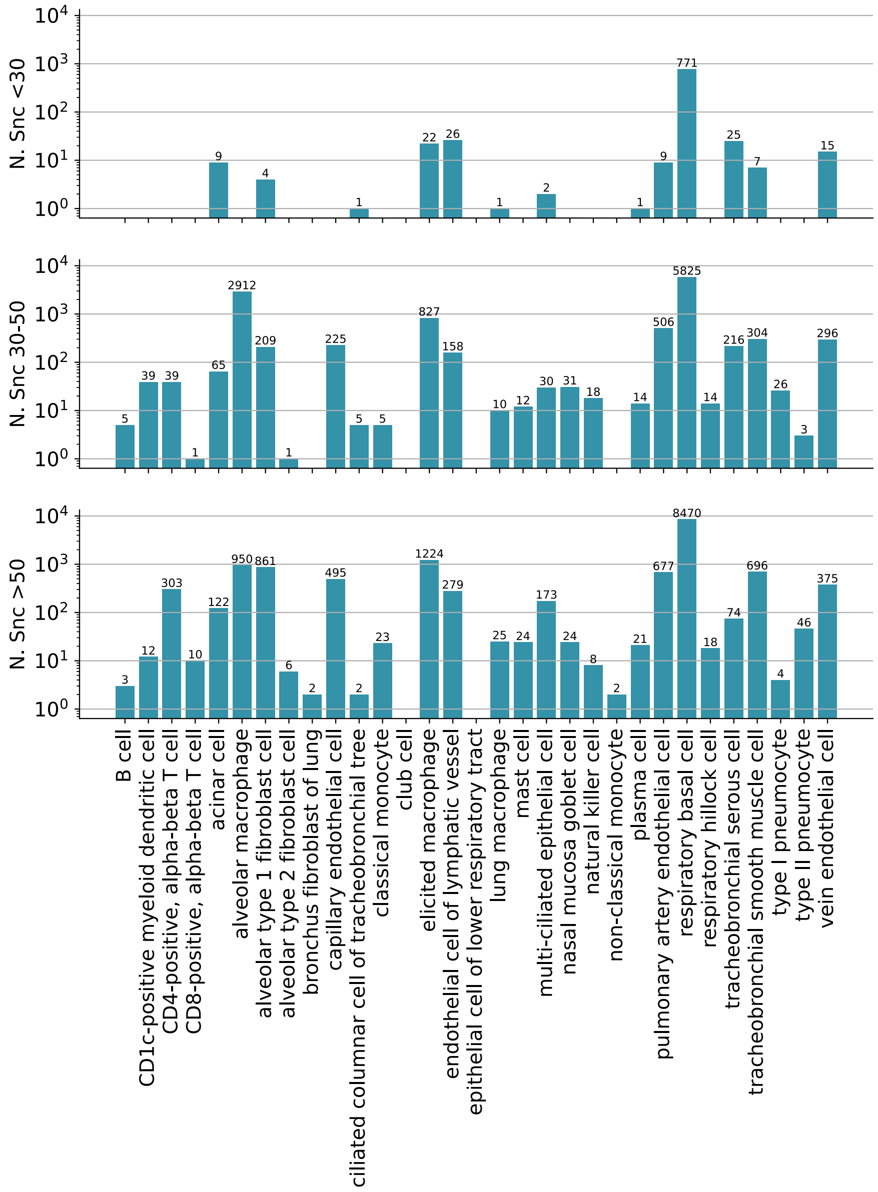

Figure EV3. PUc-predicted senescent cells by age group in the HLCA.

Bar charts showing the number of PUc-predicted senescent cells per cell type across age groups. Separate panels correspond to young (top), middle-aged (middle), and older (bottom) donors.

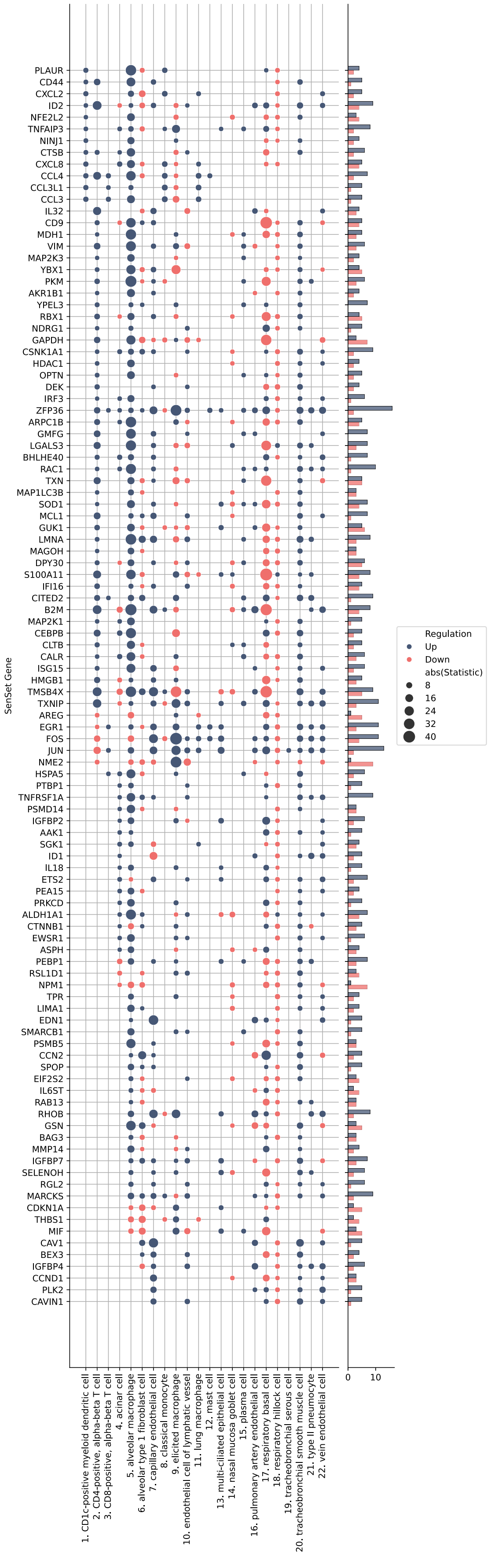
SenSet Gene
PLAUR
CD44
CXCL2
ID2
NFE2L2
TNFAIP3
NINJ1
CTSB
CXCL8
CCL4
CCL3L1
CCL3
IL32
CD9
MDH1
VIM
MAP2K3
YBX1
PKM
AKR1B1
YPEL3
RBX1
NDRG1
GAPDH
CSNK1A1
HDAC1
OPTN
DEK
IRF3
ZFP36
ARPC1B
GMFG
LGALS3
BHLHE40
RAC1
TXN
MAP1LC3B
SOD1
MCL1
GUK1
LMNA
MAGOH
DPY30
S100A11
IFI16
CITED2
B2M
MAP2K1
CEBPB
CLTB
CALR
ISG15
HMGB1
TMSB4X
TXNIP
AREG
EGR1
FOS
JUN
NME2
HSPA5
PTBP1
TNFRSF1A
PSMD14
IGFBP2
AAK1
SGK1
ID1
IL18
ETS2
PEA15
PRKCD
ALDH1A1
CTNNB1
EWSR1
ASPH
PEBP1
RSL1D1
NPM1
TPR
LIMA1
EDN1
SMARCB1
PSMB5
CCN2
SPOP
EIF2S2
IL6ST
RAB13
RHOB
GSN
BAG3
MMP14
IGFBP7
SELENOH
RGL2
MARCKS
CDKN1A
THBS1
MIF
CAV1
BEX3
IGFBP4
CCND1
PLK2
CAVIN1
Regulation
Up
Down
abs(Statistic)
8
16
24
32
40
0
10
1. CD1c-positive myeloid dendritic cell
2. CD4-positive, alpha-beta T cell
3. CD8-positive, alpha-beta T cell
4. acinar cell
5. alveolar macrophage
6. alveolar type 1 fibroblast cell
7. capillary endothelial cell
8. classical monocyte
9. elicited macrophage
10. endothelial cell of lymphatic vessel
11. lung macrophage
12. mast cell
13. multi-ciliated epithelial cell
14. nasal mucosa goblet cell
15. plasma cell
16. pulmonary artery endothelial cell
17. respiratory basal cell
18. respiratory hillock cell
19. tracheobronchial serous cell
20. tracheobronchial smooth muscle cell
21. type II pneumocyte
22. vein endothelial cell

 © The Author(s)

Figure EV4. Global enrichment of SenSet genes across lung cell types.

Heatmap showing Wilcoxon rank-sum test statistics (absolute values) for each SenSet gene (rows) across 22 lung cell types in the HLCA (columns). Color indicates direction of regulation: upregulated (blue) or downregulated (red). Source data are available online for this figure.

© The Author(s)

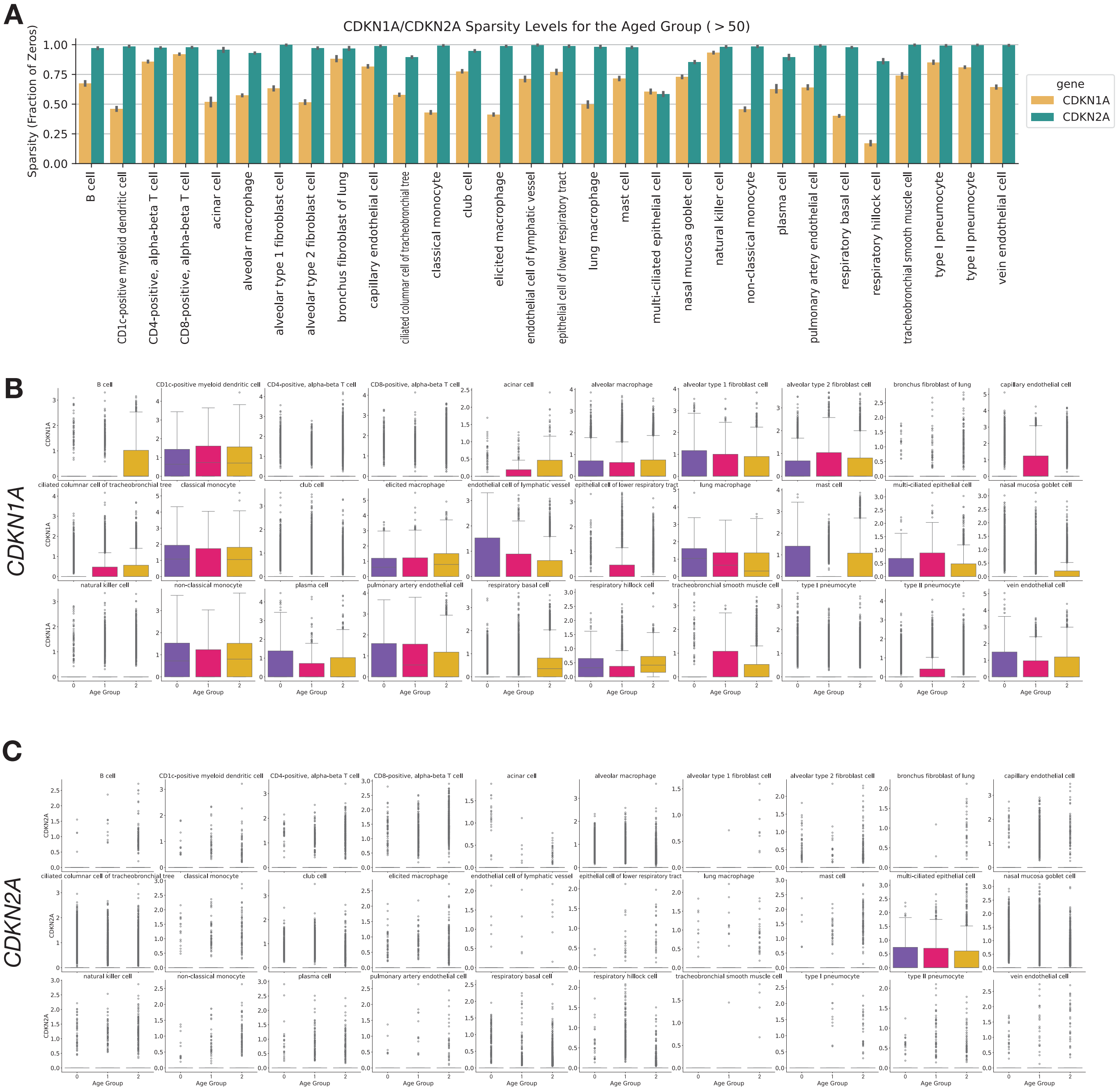

Figure EV5. CDKN1A and CDKN2A expression across cell types and age groups in the HLCA.

(**A**) Sparsity profiles showing the fraction of cells with zero expression of CDKN1A and CDKN2A across lung cell types in the oldest donor cohort. (**B**) Boxplots of normalized CDKN1A expression by cell type, stratified by young, middle-aged, and older donors. (**C**) Boxplots of normalized CDKN2A expression by cell type, stratified by the same age groups. Values of *n* shown in Figs. EV2 and 3. Boxplots show median, interquartile range, and whiskers at 1.5× IQR.

 © The Author(s)

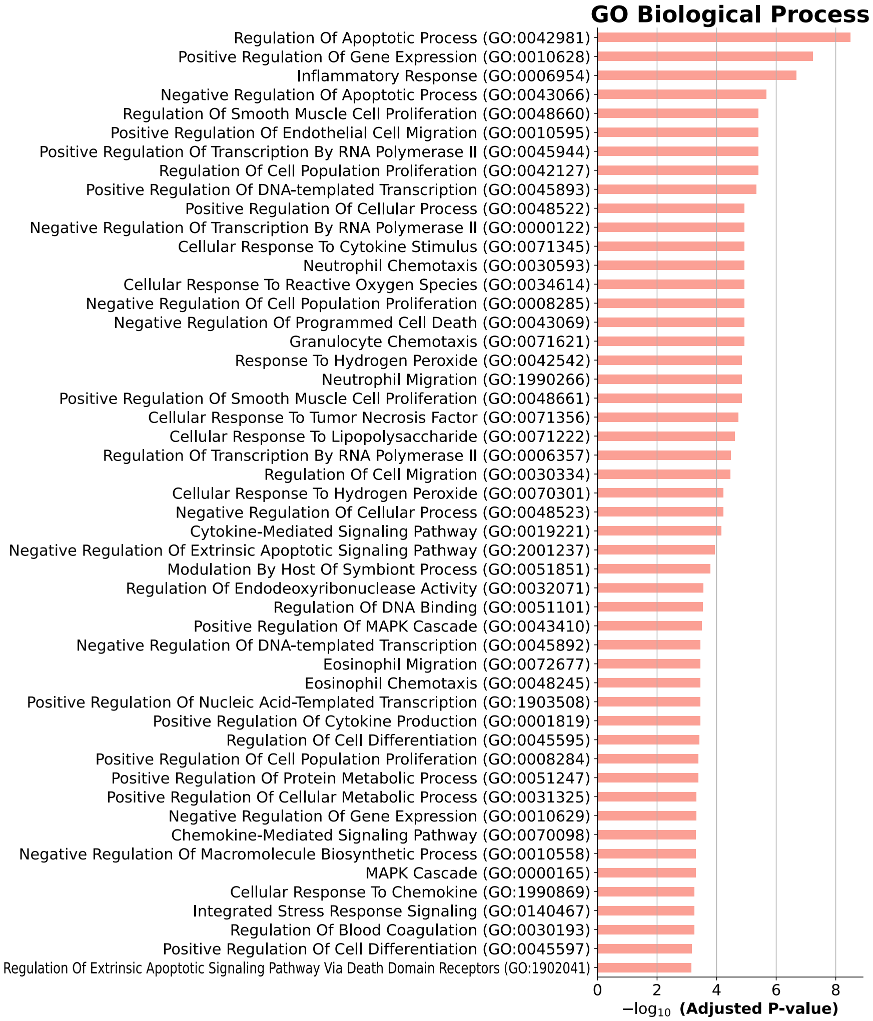

Figure EV6. Functional enrichment of SenSet genes.

Top 50 Gene Ontology (GO) terms enriched among SenSet genes. Adjusted *P* values were obtained using a hypergeometric test as implemented in the GSEApy enrichment module. Source data are available online for this figure.

© The Author(s)

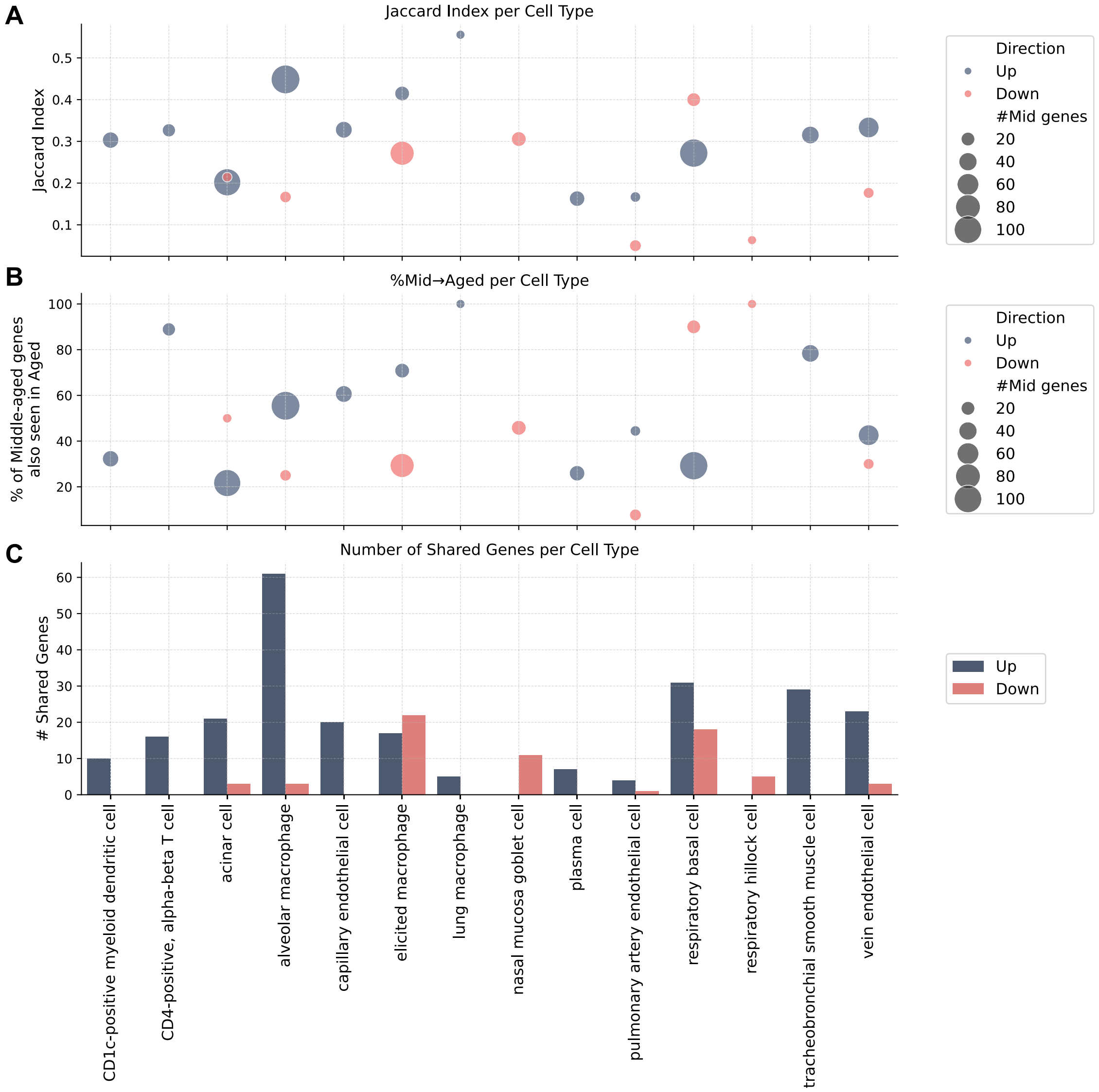

Figure EV7. Consistency of marker genes between middle-aged and older donors.

(**A**) Jaccard index per cell type comparing marker gene sets from middle-aged and older donor cohorts. (**B**) Fraction of middle-aged cohort markers also present in the older cohort. (**C**) Absolute number of shared marker genes between the two age groups. Source data are available online for this figure.

 © The Author(s)

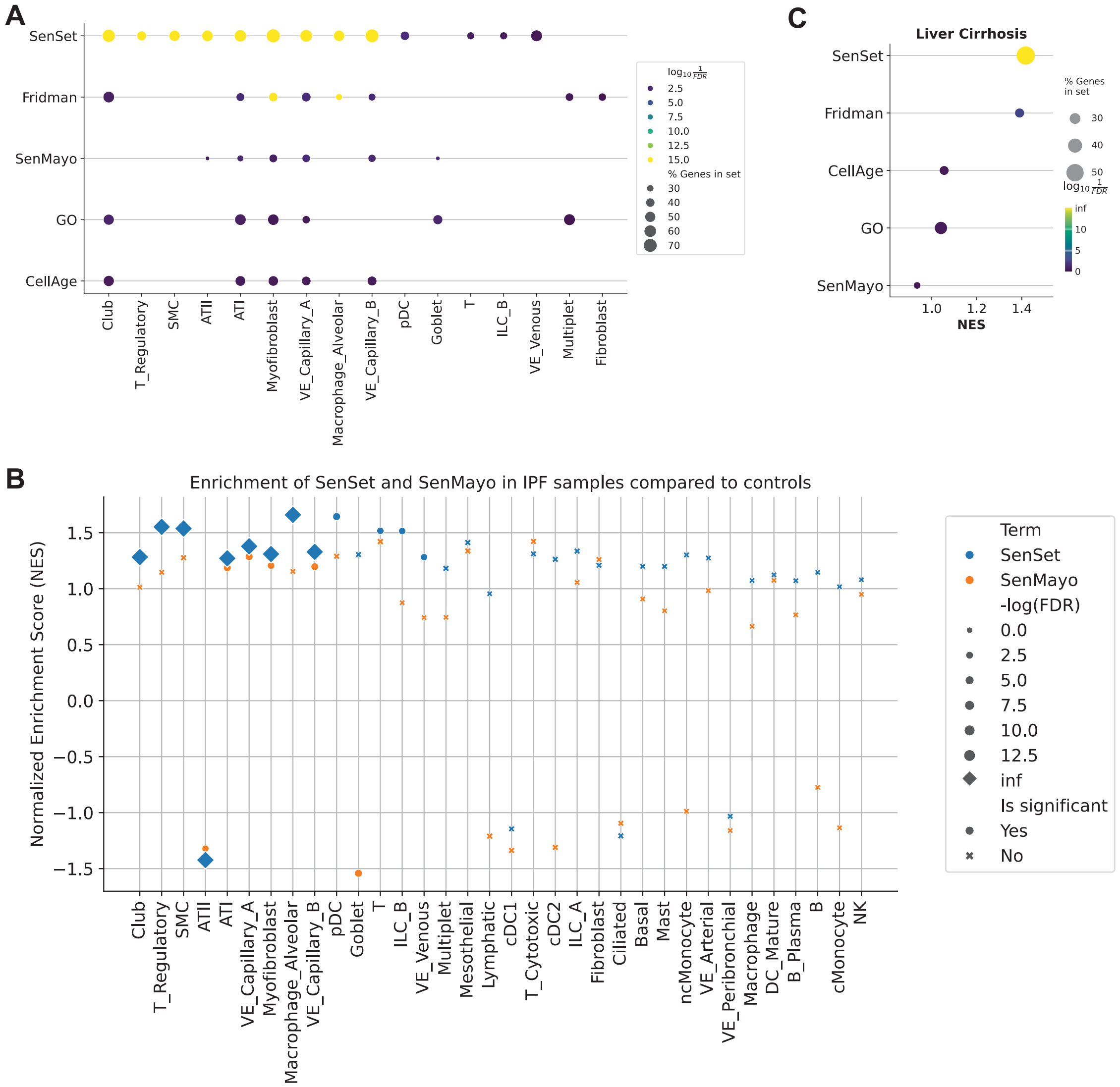

Figure EV8. Preranked GSEA in IPF and liver cirrhosis cohorts.

(**A**) Dot plot showing preranked enrichment results for five senescence gene lists in the idiopathic pulmonary fibrosis (IPF) dataset. (**B**) Comparative dot plot of normalized enrichment scores (NES) for SenSet versus SenMayo in IPF; diamonds indicate FDR = 0. (**C**) Preranked enrichment results for the liver cirrhosis dataset, highlighting SenSet enrichment. Source data are available online for this figure.

© The Author(s)

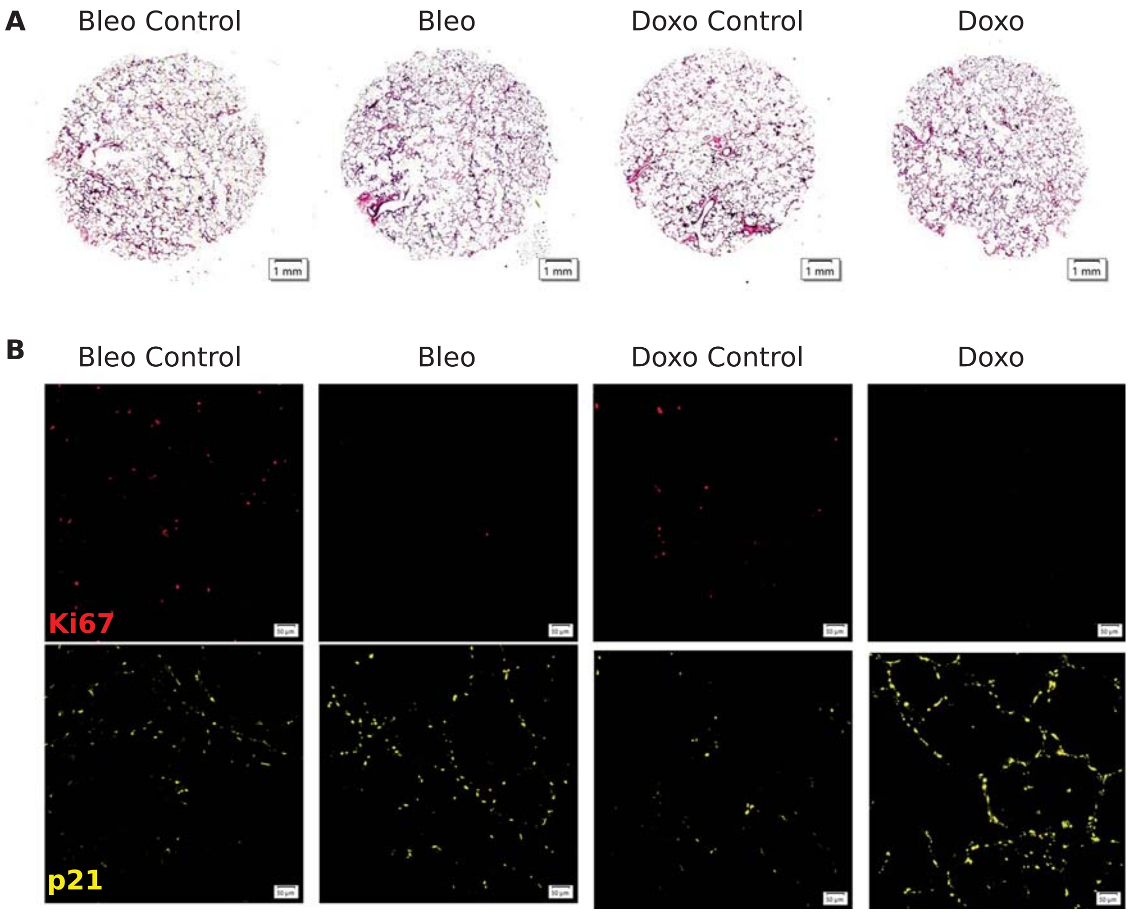

Figure EV9. Images from hPCLS treated with bleomycin and doxorubicin for 6 days.

(**A**) Hematoxylin and eosin staining of 4 µm formalin-fixed paraffin-embedded human precision-cut lung slices (PCLS) at day 6. (**B**) Immunohistofluorescence staining for p21 (yellow) and Ki67 (red) on 4-µm PCLS sections at day 6. Source data are available online for this figure.

 © The Author(s)

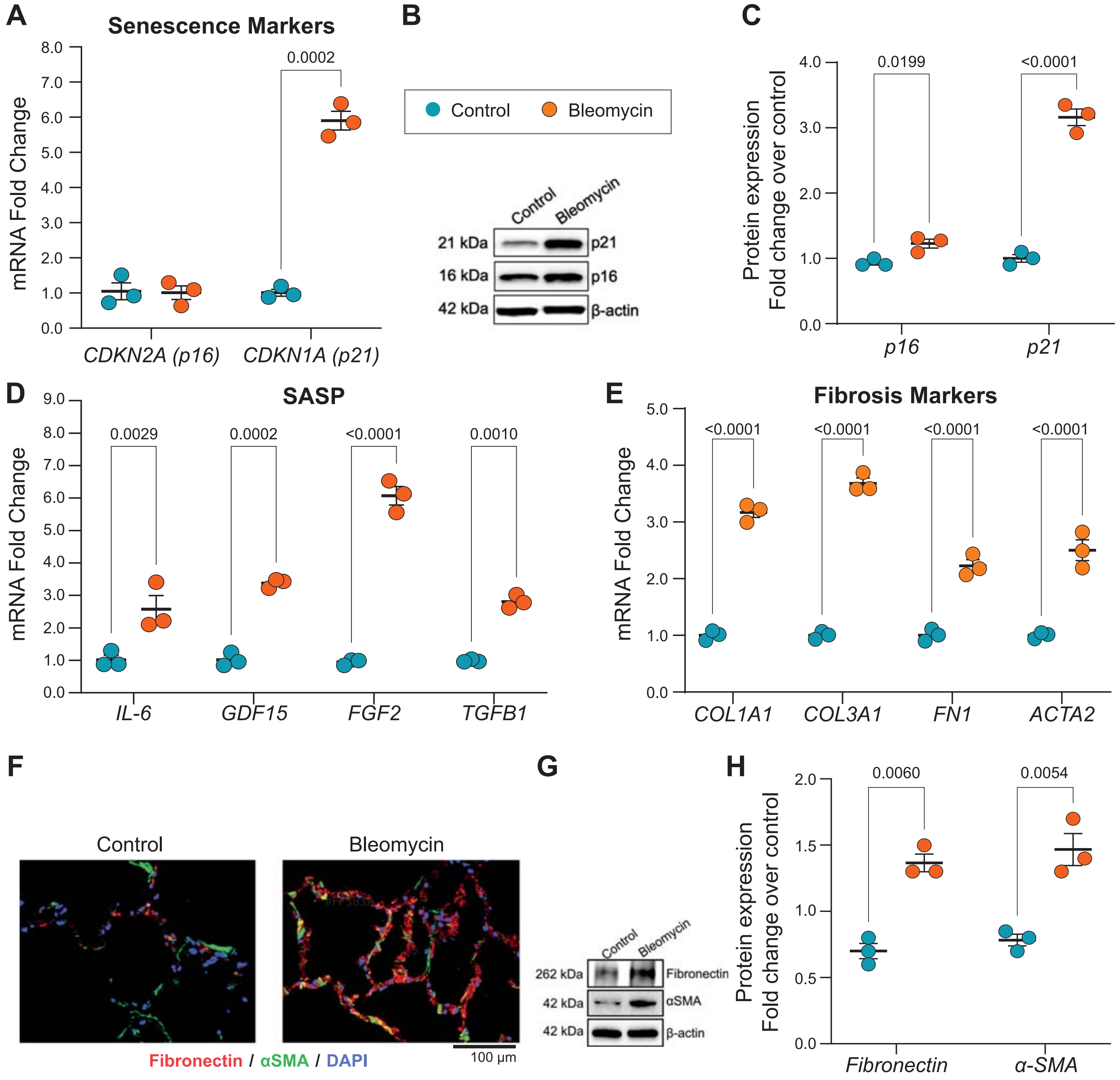

Figure EV10. Validation of senescence and fibrotic responses in bleomycin-treated samples.

(**A**) mRNA fold change of CDKN1A and CDKN2A relative to untreated controls ($n = 3$). (**B**) Representative Western blot for p21 and p16 in control versus bleomycin-treated samples. (**C**) Quantification of p16 and p21 protein levels normalized to control ($n = 3$). (**D**) mRNA fold change of key SASP factors following bleomycin exposure ($n = 3$). (**E**) mRNA fold change of fibrosis-associated genes relative to control ($n = 3$). (**F**) Immunofluorescence images comparing fibronectin (red) and α-SMA (green) in control versus bleomycin-treated cells. (**G**) Western blot analysis of fibronectin and α-SMA protein expression in control and bleomycin conditions. (**H**) Quantification of fibronectin and α-SMA protein fold change normalized to control ($n = 3$). All P values were obtained using a two-way ANOVA followed by Tukey's post hoc test ($P < 0.005$). All error bars represent the standard error of the mean (SEM). Source data are available online for this figure.

© The Author(s)

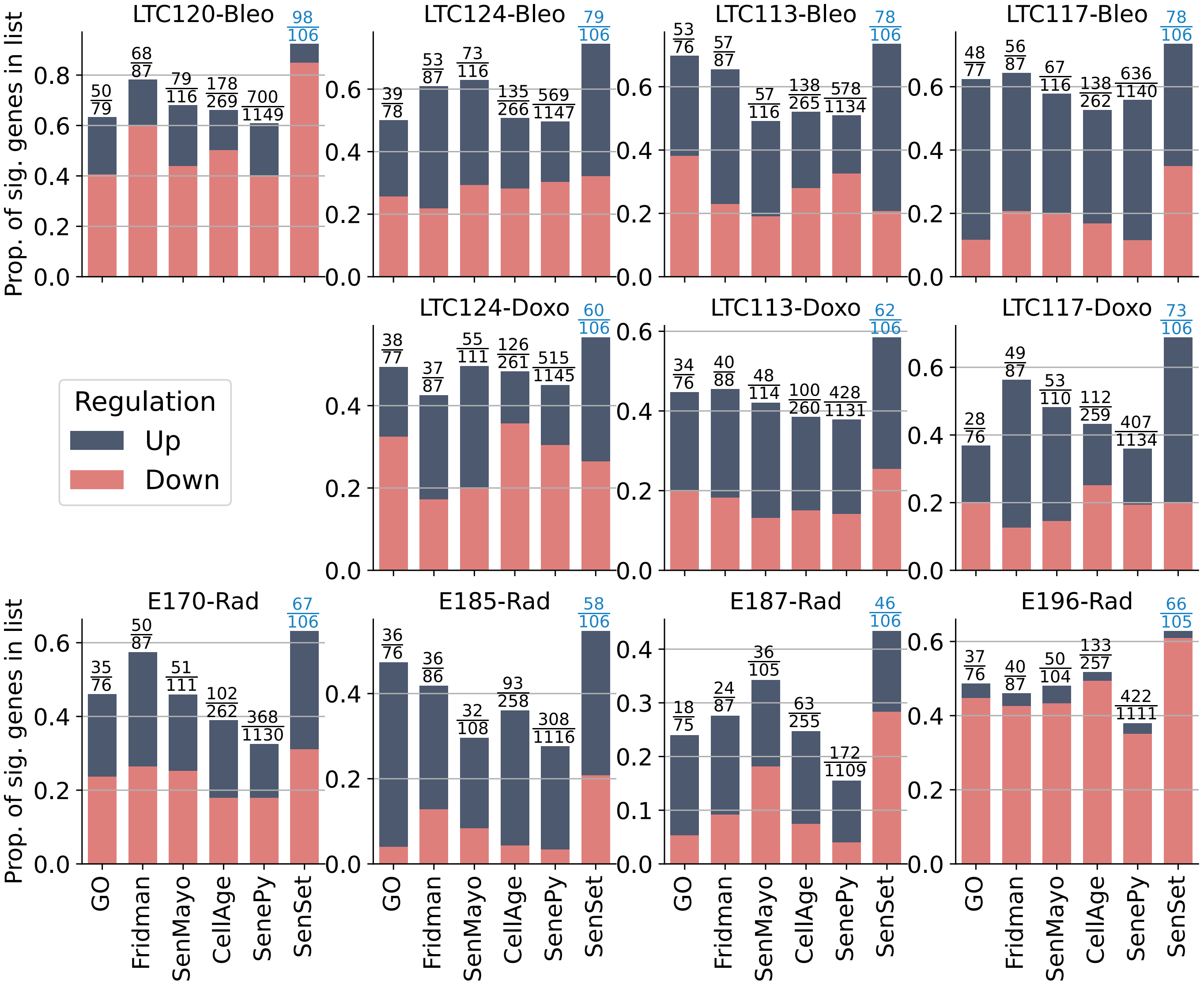

Figure EV11. SenePy comparison of senescence signatures.

Per-donor regulation fractions across senescence gene signatures. The *y* axis indicates the fraction of genes in each signature with significant expression changes. Colored segments denote upregulated (blue) and downregulated (red) genes following treatment. Source data are available online for this figure.

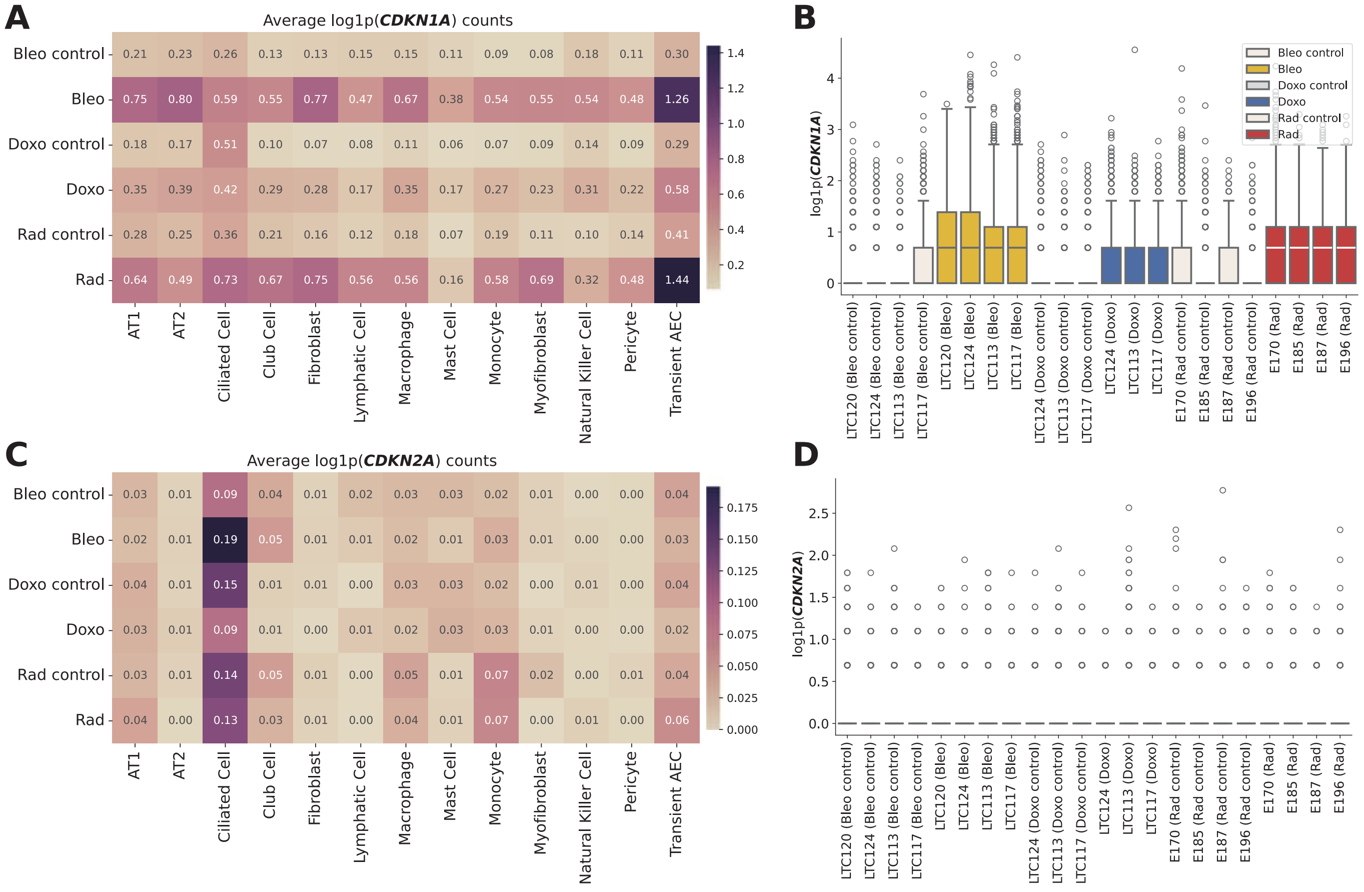

Figure EV12. CDKN1A and CDKN2A expression in PCLS by cell type and sample.

(**A**) Heatmap of mean log1p-normalized CDKN1A expression across experimental conditions and cell types. (**B**) Boxplots showing individual CDKN1A expression values per subject and condition. Values of *n* shown in Tables 3 and 4. Boxplots show median, interquartile range, and whiskers at 1.5× IQR. (**C**) Heatmap of mean log1p-normalized CDKN2A expression across the same conditions and cell types. (**D**) Boxplots showing individual CDKN2A expression values, matching the layout in (**B**).

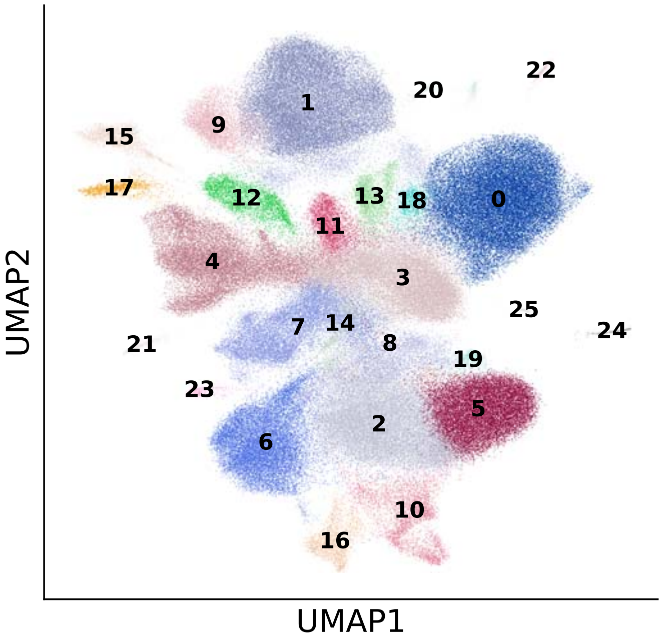

Figure EV13. Clustering of integrated PCLS data.

UMAP visualization showing all clusters identified using the Leiden clustering method in Scanpy.

 © The Author(s)

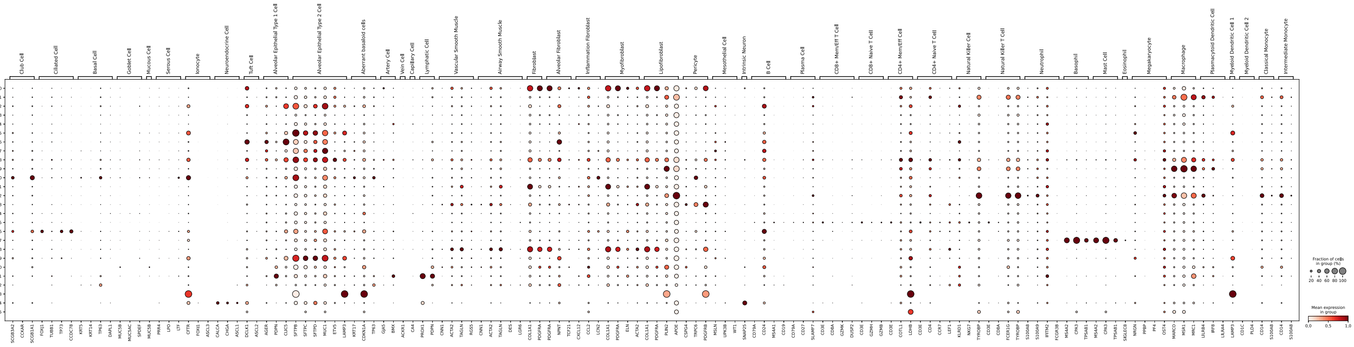

Figure EV14. Canonical markers used to identify PCLS cell types.

Dot plot showing canonical marker genes used for cell type annotation in the PCLS dataset. Markers were derived from Travaglini et al and supplemented with additional markers for transient alveolar epithelial cells. Source data are available online for this figure.

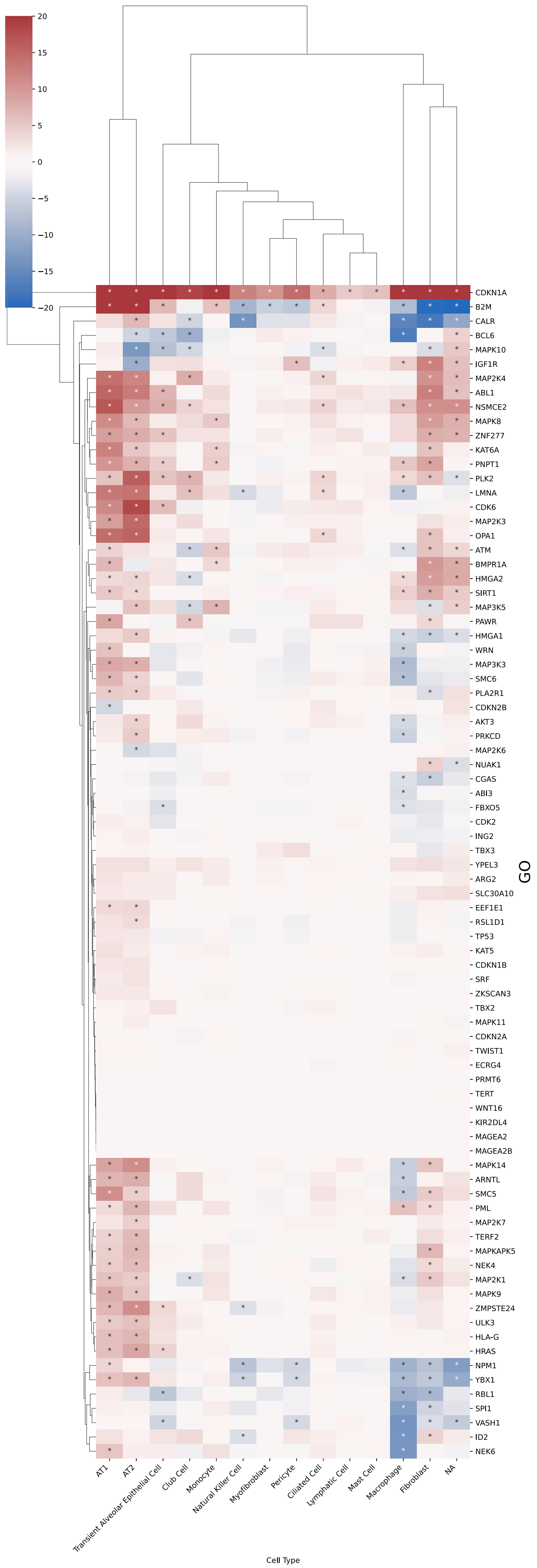
20
15
10
5
0
−5
−10
−15
−20
CDKN1A
B2M
CALR
BCL6
MAPK10
IGF1R
MAP2K4
ABL1
NSMCE2
MAPK8
ZNF277
KAT6A
PNPT1
PLK2
LMNA
CDK6
MAP2K3
OPA1
ATM
BMPR1A
HMGA2
SIRT1
MAP3K5
PAWR
HMGA1
WRN
MAP3K3
SMC6
PLA2R1
CDKN2B
AKT3
PRKCD
MAP2K6
NUAK1
CGAS
ABI3
FBXO5
CDK2
ING2
TBX3
YPEL3
ARG2
SLC30A10
EEF1E1
RSL1D1
TP53
KAT5
CDKN1B
SRF
ZKSCAN3
TBX2
MAPK11
CDKN2A
TWIST1
ECRG4
PRMT6
TERT
WNT16
KIR2DL4
MAGEA2
MAGEA2B
MAPK14
ARNTL
SMC5
PML
MAP2K7
TERF2
MAPKAPK5
NEK4
MAP2K1
MAPK9
ZMPSTE24
ULK3
HLA-G
HRAS
NPM1
YBX1
RBL1
SPI1
VASH1
ID2
NEK6
GO
AT1
AT2
Transient Alveolar Epithelial Cell
Club Cell
Monocyte
Natural Killer Cell
Myofibroblast
Pericyte
Ciliated Cell
Lymphatic Cell
Mast Cell
Macrophage
Fibroblast
NA
Cell Type

 © The Author(s)

◀ **Figure EV15. Regulation of GO marker genes in the PCLS model.**

Heatmap showing regulation of Gene Ontology-derived marker genes across cell types in treated versus control PCLS samples. Source data are available online for this figure.

© The Author(s)

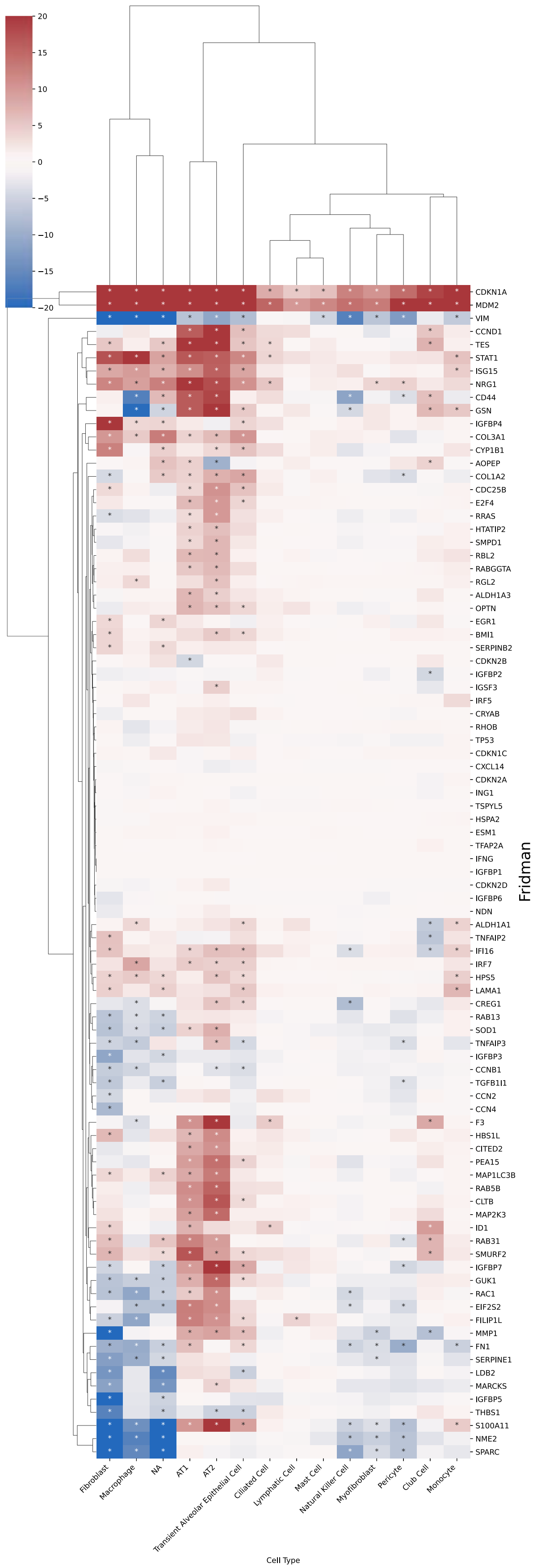
20
15
10
5
0
−5
−10
−15
−20
CDKN1A
MDM2
VIM
CCND1
TES
STAT1
ISG15
NRG1
CD44
GSN
IGFBP4
COL3A1
CYP1B1
AOPEP
COL1A2
CDC25B
E2F4
RRAS
HTATIP2
SMPD1
RBL2
RABGGTA
RGL2
ALDH1A3
OPTN
EGR1
BMI1
SERPINB2
CDKN2B
IGFBP2
IGSF3
IRF5
CRYAB
RHOB
TP53
CDKN1C
CXCL14
CDKN2A
ING1
TSPYL5
HSPA2
ESM1
TFAP2A
IFNG
IGFBP1
CDKN2D
IGFBP6
NDN
ALDH1A1
TNFAIP2
IFI16
IRF7
HPS5
LAMA1
CREG1
RAB13
SOD1
TNFAIP3
IGFBP3
CCNB1
TGFB1I1
CCN2
CCN4
F3
HBS1L
CITED2
PEA15
MAP1LC3B
RAB5B
CLTB
MAP2K3
ID1
RAB31
SMURF2
IGFBP7
GUK1
RAC1
EIF2S2
FILIP1L
MMP1
FN1
SERPINE1
LDB2
MARCKS
IGFBP5
THBS1
S100A11
NME2
SPARC
Fridman
Fibroblast
Macrophage
NA
AT1
AT2
Transient Alveolar Epithelial Cell
Ciliated Cell
Lymphatic Cell
Mast Cell
Natural Killer Cell
Myofibroblast
Pericyte
Club Cell
Monocyte
Cell Type

 © The Author(s)

Figure EV16. Regulation of Fridman marker genes in the PCLS model.

Heatmap showing regulation of Fridman senescence-associated marker genes across cell types in treated versus control PCLS samples. Source data are available online for this figure.

© The Author(s)

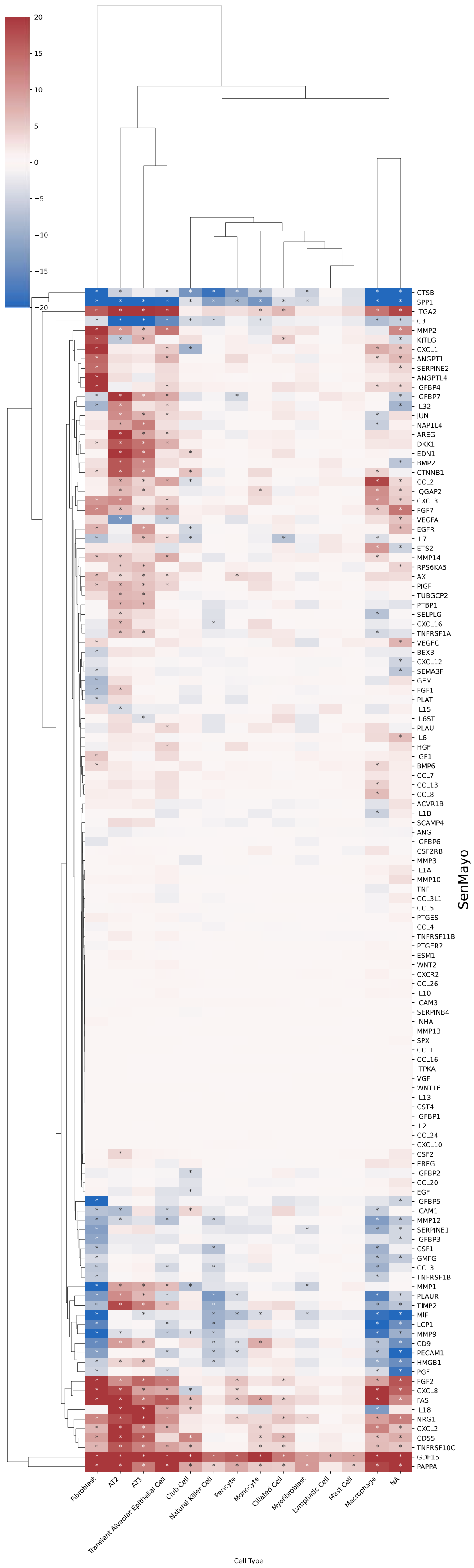
20
15
10
5
0
−5
−10
−15
−20
CTSB
SPP1
ITGA2
C3
MMP2
KITLG
CXCL1
ANGPT1
SERPINE2
ANGPTL4
IGFBP4
IGFBP7
IL32
JUN
NAP1L4
AREG
DKK1
EDN1
BMP2
CTNNB1
CCL2
IQGAP2
CXCL3
FGF7
VEGFA
EGFR
IL7
ETS2
MMP14
RPS6KA5
AXL
PIGF
TUBGCP2
PTBP1
SELPLG
CXCL16
TNFRSF1A
VEGFC
BEX3
CXCL12
SEMA3F
GEM
FGF1
PLAT
IL15
IL6ST
PLAU
IL6
HGF
IGF1
BMP6
CCL7
CCL13
CCL8
ACVR1B
IL1B
SCAMP4
ANG
IGFBP6
CSF2RB
MMP3
IL1A
MMP10
TNF
CCL3L1
CCL5
PTGES
CCL4
TNFRSF11B
PTGER2
ESM1
WNT2
CXCR2
CCL26
IL10
ICAM3
SERPINB4
INHA
MMP13
SPX
CCL1
CCL16
ITPKA
VGF
WNT16
IL13
CST4
IGFBP1
IL2
CCL24
CXCL10
CSF2
EREG
IGFBP2
CCL20
EGF
IGFBP5
ICAM1
MMP12
SERPINE1
IGFBP3
CSF1
GMFG
CCL3
TNFRSF1B
MMP1
PLAUR
TIMP2
MIF
LCP1
MMP9
CD9
PECAM1
HMGB1
PGF
FGF2
CXCL8
FAS
IL18
NRG1
CXCL2
CD55
TNFRSF10C
GDF15
PAPPA
SenMayo
Fibroblast
AT2
AT1
Transient Alveolar Epithelial Cell
Club Cell
Natural Killer Cell
Pericyte
Monocyte
Ciliated Cell
Myofibroblast
Lymphatic Cell
Mast Cell
Macrophage
NA
Cell Type

 © The Author(s)

Figure EV17. Regulation of SenMayo marker genes in the PCLS model.

Heatmap showing regulation of SenMayo marker genes across cell types in treated versus control PCLS samples. Source data are available online for this figure.

© The Author(s)

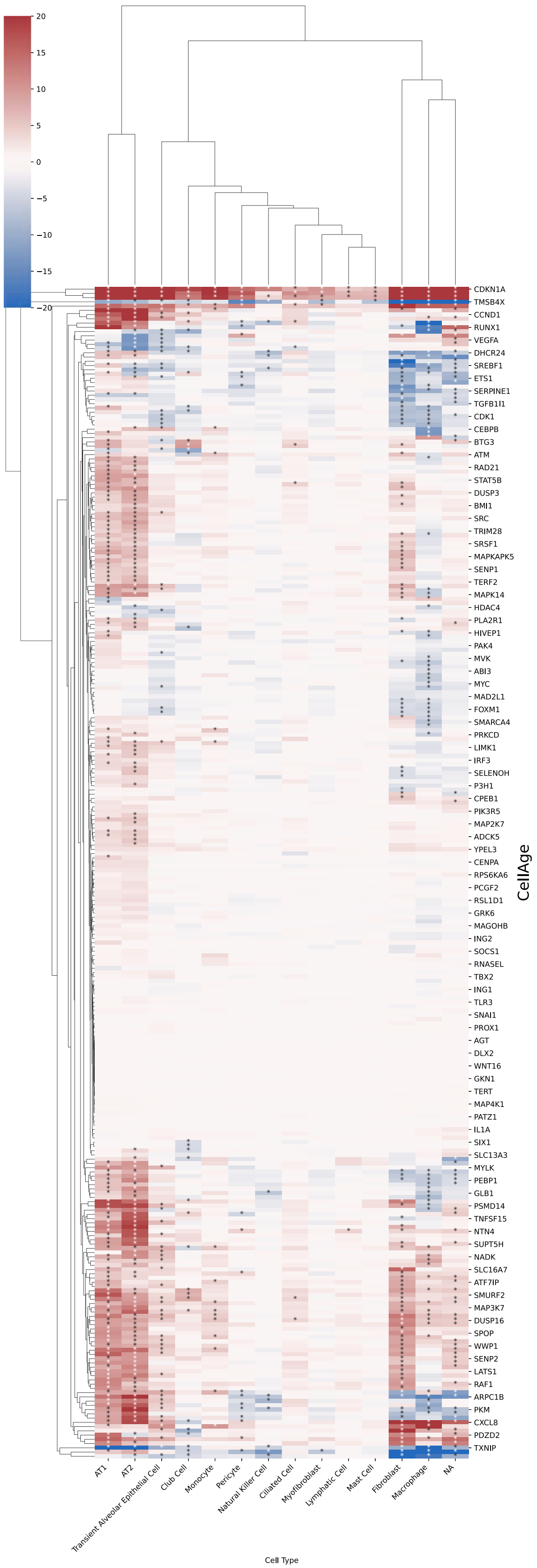
20
15
10
5
0
−5
−10
−15
−20
CDKN1A
TMSB4X
CCND1
RUNX1
VEGFA
DHCR24
SREBF1
ETS1
SERPINE1
TGFB1I1
CDK1
CEBPB
BTG3
ATM
RAD21
STAT5B
DUSP3
BMI1
SRC
TRIM28
SRSF1
MAPKAPK5
SENP1
TERF2
MAPK14
HDAC4
PLA2R1
HIVEP1
PAK4
MVK
ABI3
MYC
MAD2L1
FOXM1
SMARCA4
PRKCD
LIMK1
IRF3
SELENOH
P3H1
CPEB1
PIK3R5
MAP2K7
ADCK5
YPEL3
CENPA
RPS6KA6
PCGF2
RSL1D1
GRK6
MAGOHB
ING2
SOCS1
RNASEL
TBX2
ING1
TLR3
SNAI1
PROX1
AGT
DLX2
WNT16
GKN1
TERT
MAP4K1
PATZ1
IL1A
SIX1
SLC13A3
MYLK
PEBP1
GLB1
PSMD14
TNFSF15
NTN4
SUPT5H
NADK
SLC16A7
ATF7IP
SMURF2
MAP3K7
DUSP16
SPOP
WWP1
SENP2
LATS1
RAF1
ARPC1B
PKM
CXCL8
PDZD2
TXNIP
CellAge
AT1
AT2
Transient Alveolar Epithelial Cell
Club Cell
Monocyte
Pericyte
Natural Killer Cell
Ciliated Cell
Myofibroblast
Lymphatic Cell
Mast Cell
Fibroblast
Macrophage
NA
Cell Type

 © The Author(s)

Figure EV18. Regulation of CellAge marker genes in the PCLS model.

Heatmap showing regulation of CellAge marker genes across cell types in treated versus control PCLS samples. Source data are available online for this figure.

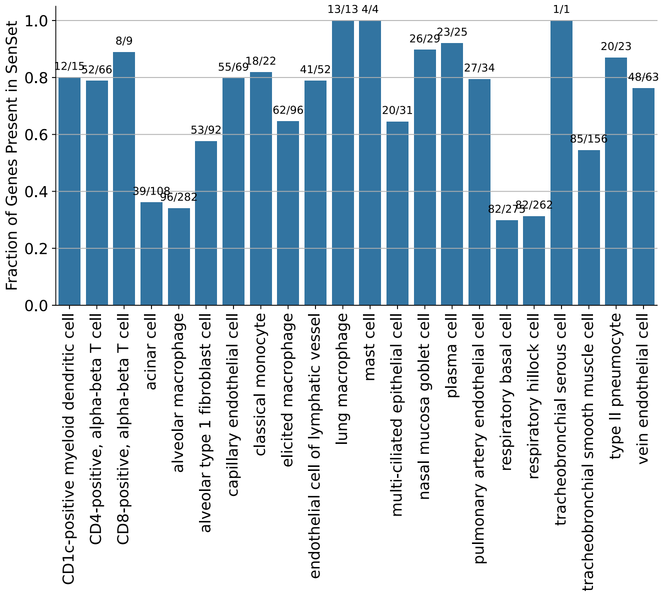

Figure EV19. Fraction of cell-type-derived markers retained in SenSet.

Bar plot showing the proportion of cell-type-specific marker genes that were ultimately included in the SenSet signature. Source data are available online for this figure.

 © The Author(s)
